# Lipopeptide antibiotics disrupt interactions of undecaprenyl phosphate with UptA

**DOI:** 10.1073/pnas.2408315121

**Published:** 2024-10-03

**Authors:** Abraham O. Oluwole, Neha V. Kalmankar, Michela Guida, Jack L. Bennett, Giovanna Poce, Jani R. Bolla, Carol V. Robinson

**Affiliations:** ^a^Department of Chemistry, University of Oxford, Oxford OX1 3QZ, United Kingdom; ^b^The Kavli Institute for Nanoscience Discovery, University of Oxford, Oxford OX1 3QU, United Kingdom; ^c^Department of Chemistry and Technologies of Drug, Sapienza University of Rome, Rome 00185, Italy; ^d^Department of Biology, University of Oxford, Oxford OX1 3RB, United Kingdom

**Keywords:** peptidoglycan, undecaprenyl phosphate transporter A, UptA, native mass spectrometry

## Abstract

The peptidoglycan pathway represents one of the most successful antibacterial targets with the last critical step being the flipping of carrier lipid, undecaprenyl phosphate (C_55_-P), across the membrane to reenter the pathway. This translocation of C_55_-P is facilitated by DedA and DUF368 domain-containing family membrane proteins via unknown mechanisms. Here, we employ native mass spectrometry to investigate the interactions of UptA, a member of the DedA family of membrane protein from *Bacillus subtilis*, with C_55_-P, membrane phospholipids, and cell wall-targeting antibiotics. Our results show that UptA, expressed and purified in *Escherichia coli*, forms monomer–dimer equilibria, and binds to C_55_-P in a pH-dependent fashion. Specifically, we show that UptA interacts more favorably with C_55_-P over shorter-chain analogs and membrane phospholipids. Moreover, we demonstrate that lipopeptide antibiotics, amphomycin and aspartocin D, can directly inhibit UptA function by out-competing the substrate for the protein binding, in addition to their propensity to form complex with free C_55_-P. Overall, this study shows that UptA-mediated translocation of C_55_-P is potentially mediated by pH and anionic phospholipids and provides insights for future development of antibiotics targeting carrier lipid recycling.

## Significance

Peptidoglycan polymer is the core structural element of bacterial cell walls, and its biosynthesis depends on precursor transfer from the cytosol into the periplasm by undecaprenyl phosphate (C_55_-P). This molecule needs to be flipped across the membrane to reenter the pathway by the UptA-type and PopT-type membrane transporters, but how this process occurs is currently unknown. Using cutting-edge mass spectrometry approaches, we provide molecular-level insights into the interplay of UptA with C_55_-P, phospholipids, and antibiotics. We established the substrate specificity of UptA and provided evidence for an additional mode of action for amphomycin and derivatives, consisting of direct physical interaction with UptA to destabilize C_55_-P binding, offering avenues for antibiotic development targeting the bacterial cell wall.

**Fig. 1.**
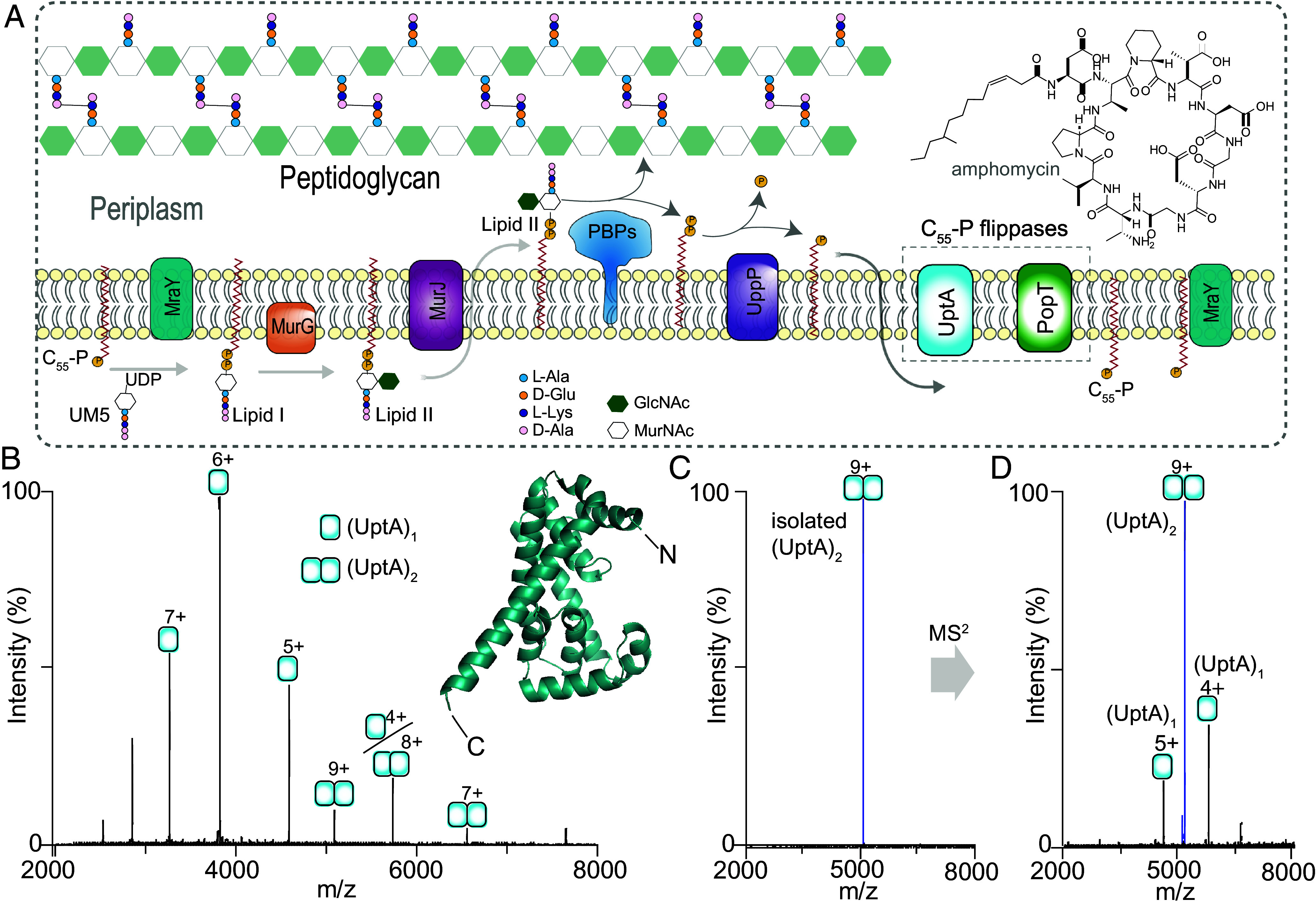
UptA is primarily monomeric with a low population of dimer. (*A*) Schematic depiction of the lipid II cycle of peptidoglycan biosynthesis. C_55_-P, Undecaprenyl monophosphate; C_55_-PP, Undecaprenyl diphosphate; UM5, UDP-*N*-acetylmuramyl-pentapeptide; PBPs, penicillin-binding proteins. (*B*) Native mass spectrum of UptA (2.2 µM) liberated from a buffer containing 200 mM ammonium acetate (pH 8.0), 0.05% LDAO. Peaks are assigned to UptA in monomeric and dimeric forms. Insert, the model structure of UptA (AFO31823-F1) predicted by AlphaFold (22). Predicted N- and C- termini are indicated. (*C*) Quadrupole isolation of the UptA dimer (9+ charge state). (*D*) MS/MS of the isolated UptA dimer upon collisional activation (190 V). The dimer dissociates into individual UptA protomers confirming their noncovalent association.

The global threat of drug-resistant bacterial pathogens calls for renewed efforts in the search for new antibiotics, along with the identification of new biochemical pathways and interactions that can be effectively inhibited. A key antibiotic target is peptidoglycan, the cell wall polymer that provides bacteria with resistance to osmotic stress and environmental assaults (1, 2). Accordingly, topline antibiotics such as amoxicillin and vancomycin (3, 4) inhibit the transpeptidation step of the peptidoglycan synthesis (5–7). Moreover, promising antibiotic candidates, such as ramoplanin and moenomycin, target the transglycosylation step (8, 9). Peptidoglycan biosynthesis begins with the formation of uridine diphosphate-*N*-acetylmuramyl-pentapeptide (UM5) in the cytosol (10), followed by its coupling to the lipid carrier undecaprenyl monophosphate (C_55_-P) on the cytosolic side of the cytoplasmic membrane (11, 12). The resulting lipid I is further decorated with a GlcNAc residue to form lipid II (13) before being flipped across the cytoplasmic membrane (7, 14, 15). In the periplasm, the headgroup of lipid II is polymerized and cross-linked into the existing meshwork (16–18). The lipid carrier is then released as undecaprenyl diphosphate (C_55_-PP). To participate in the next round of precursor transfer, C_55_-PP must be dephosphorylated to form C_55_-P (19–21) and then flipped across the cytoplasmic membrane such that its phosphate headgroup returns to the cytoplasmic side. The mechanism by which C_55_-P is translocated across the cytoplasmic membrane is the least understood of the membrane-associated steps of the peptidoglycan pathway (Fig. 1*A*).

Recently, proteins belonging to the DedA- and the DUF368 domain-containing families were shown to facilitate trans-bilayer transport of C_55_-P in the Gram-positive bacteria *Bacillus subtilis* and *Staphylococcus aureus* (23), and in the Gram-negative bacteria *Vibrio cholerae* (24). DedA proteins are highly conserved, having 8 homologs in the model bacteria *Escherichia coli* and 6 homologs in *B. subtilis* (25–27). Members of the DedA transporters in *B. subtilis* include UptA and PetA, which are proposed to facilitate trans-bilayer flipping of C_55_-P and phosphatidylethanolamine, respectively (23, 28). Compared to the DedA family, the DUF368-domain containing proteins, exemplified by PopT, are less conserved, being found in *S. aureus* and *V. cholerae,* but are absent in *B. subtilis* and *E. coli* (29). *V. cholerae* PopT becomes important for cell survival only under alkaline conditions (24). Several Gram-positive and Gram-negative bacteria encode one or multiple UptA-type transporters suggesting that the C_55_-P recycling process, mediated by these flippases, is broadly conserved across different bacterial species. For example, the single DedA homolog in *Borrelia burgdorferi* is an essential protein required for proper cell division (30). Therefore, the recycling step of peptidoglycan biosynthesis mediated by UptA-type flippases make promising antibiotics target (31). This necessitates a molecular-level understanding of how UptA interacts with its cognate substrate, C_55_-P, and potentially with membrane phospholipids, to elucidate the mechanism of C_55_-P translocation.

Herein we employ native mass spectrometry (native MS) to elucidate how *B. subtilis* UptA interacts with multiple lipidic substrates and antibiotics. We show that purified UptA exists as an equilibrium of monomers and dimers. We further show that UptA interacts more favorably with C_55_-P than C_55_-PP and membrane phospholipids and then provide molecular-level evidence on the amino acid residues involved in C_55_-P binding. We find that UptA binds to its cognate ligand C_55_-P in a pH-dependent manner and show that lipopeptide antibiotics, such as amphomycin and aspartocin D, bind to UptA with higher affinity than other cell-wall targeting antimicrobial peptides such as bacitracin and vancomycin. Interestingly, amphomycin and its analogs outcompete the flippase UptA for C_55_-P binding, raising the possibility of an additional mode of action and avenue for the development of novel antibiotics to inhibit peptidoglycan biosynthesis.

## Results

### Monomer–Dimer Equilibrium of UptA.

We expressed *B. subtilis* UptA in *E. coli* and introduced the purified protein into the mass spectrometer from a buffer containing 0.05% LDAO and 200 mM ammonium acetate (pH 8.0). The mass spectrum of UptA displays two charge state distributions assigned to monomers (22,963.49 ± 0.70 Da) and dimers (45,925.21 ± 0.65 Da) (Fig. 1*B*). To confirm the noncovalent association of UptA monomers, we isolated the 9+ charge state assigned to the dimer and subjected these ions to high-energy collisional dissociation (HCD, Fig. 1*C*). The resulting MS^2^ spectrum displays new peaks that correspond by mass to UptA protomers and bear charges complementary to the isolated parent ions (+5 and +4) (Fig. 1*D*). Of note, the experimental mass for the UptA monomer is higher than the theoretical sequence mass (22,936.36 Da) by 28 Da, suggesting an endogenous modification. To identify this unknown modification, we activated the monomer (6+ charge state) using HCD, yielding a plethora of *b*- and *y-*type fragment ions (*SI Appendix*, Fig. S1). The observed fragments are consistent with the formylation of the N-terminal methionine of UptA (*SI Appendix*, Fig. S1). Bacterial inner membrane proteins are normally cotranslationally formylated on the N terminus; however, this modification is rarely observed in vitro due to subsequent removal by the cytosolic deformylase (32). Therefore, retainment of N-terminal formylation on UptA suggests periplasmic localization of the N terminus of UptA, thus supporting the predicted N-out topology (23). Additionally, we observe UptA in monomeric and dimeric forms in other detergents (*SI Appendix*, Fig. S2), confirming the oligomeric composition of UptA, which might apply to other bacterial DedA homologs (33).

### UptA Binds C_55_-P with High Affinity.

**Fig. 2.**
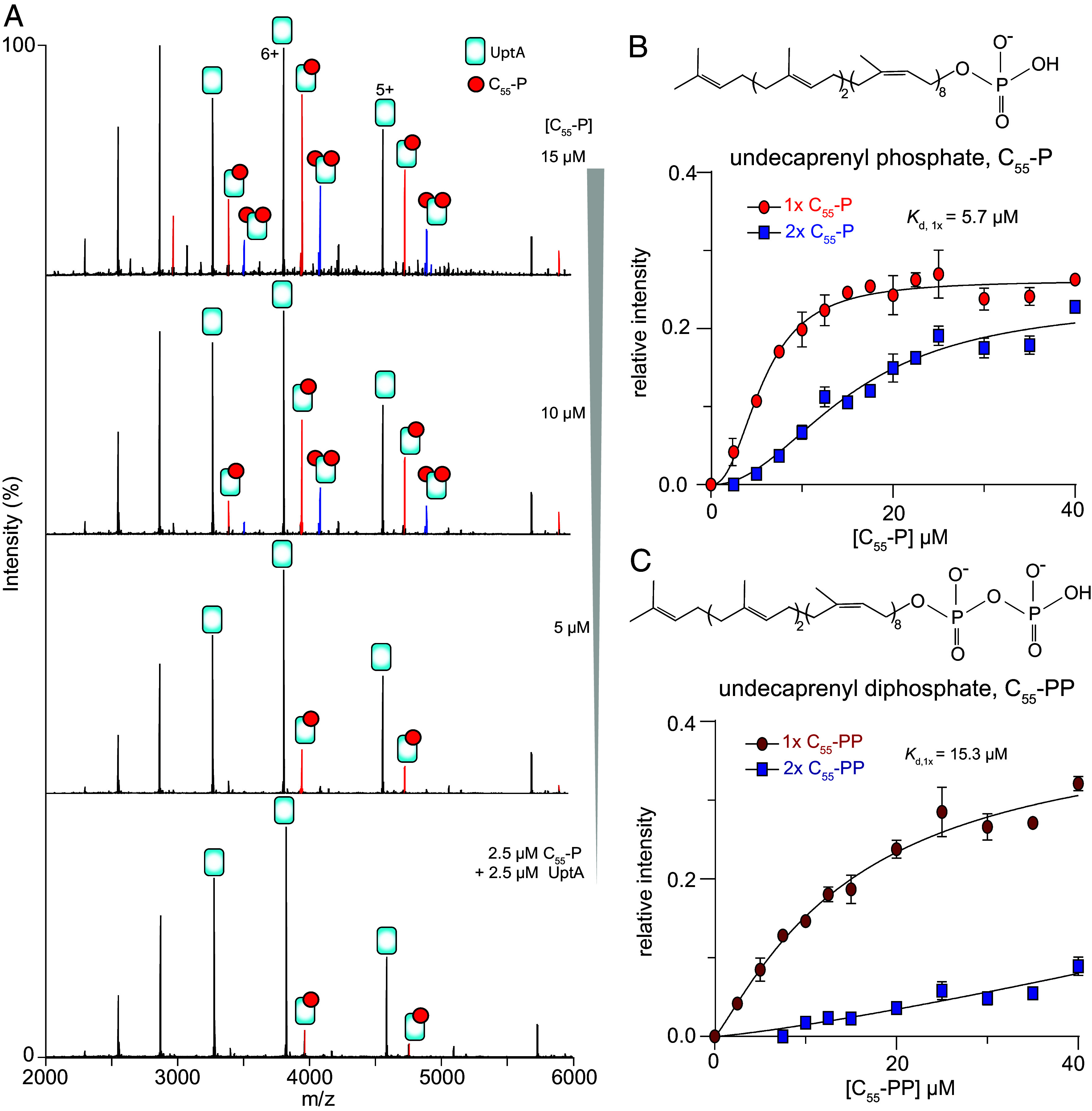
UptA binds C_55_-P with a high affinity. (*A*) Spectra for UptA incubated with different concentrations of C_55_-P in a buffer containing 200 mM ammonium acetate (pH 8), 0.05% LDAO. (*B* and *C*) The relative abundance of UptA bound to one (1×) or two (2×) molecules of C_55_-P (panel *B*) and C_55_-PP (panel *C*). Inserts, chemical structures of C_55_-P and C_55_-PP. Each data point represents an average of three replicate measurements, and the curves represent the best fits of the data to Hill’s equation (*Materials and Methods*), error bars are SD.

We explored the binding affinity of UptA toward C_55_-P by recording mass spectra for solutions containing 2.5 µM UptA and 0 to 40 µM C_55_-P in 200 mM ammonium acetate, 0.05% LDAO at pH 8.0. The mass spectrum for the equimolar mixture of UptA and C_55_-P yielded peaks corresponding to UptA in its apo form and in complex with C_55_-P (Fig. 2*A*), an indication of high-affinity binding interactions. Further increase in the concentration of C_55_-P enhanced the intensity of the UptA:C_55_-P complex, together with the emergence of additional binding events (Fig. 2*A*). We deconvoluted the spectra (34) to extract mean relative intensities of bound and unbound forms of UptA in the spectral series. The data reflected an increase in the relative abundance of UptA:(C_55_-P)_n_ complexes as a function of the concentration of C_55_-P (Fig. 2*B*). For the first binding event, the relative intensity plateaued at a protein/ligand molar ratio of ~4 (Fig. 2*B*). By fitting the experimentally observed intensity ratios to the Hill equation, we obtained an apparent *K*_d_ = 5.7 µM (95% CI: 5.0 to 6.4 µM) for the binding of one C_55_-P molecule to UptA. This *K*_d_ value for UptA/C_55_-P binding is of the same order of magnitude as the binding interactions for MurG/lipid I (*K*_d_ = 1.89 ± 0.6 µM) (35) and MurJ/lipid II (*K*_d_ = 2.9 ± 0.6 µM) (14), indicating that UptA exhibits a similarly high affinity binding with respect to C_55_-P.

To investigate the binding selectivity of UptA for monophosphate (C_55_-P) versus diphosphate (C_55_-PP) forms of the lipid carrier, we performed comparative binding assays. First, we recorded mass spectra for a mixture of UptA with increasing concentrations of C_55_-PP (*SI Appendix*, Fig. S3). We observed peaks assigned to UptA in ligand-free and C_55_-PP-bound forms, and the relative intensity of the UptA:C_55_-PP complex increased with increasing concentration of C_55_-PP (Fig. 2*C*). We then fit the data to Hill equation to obtain an apparent K_d_ for this system. The relative intensities of UptA:C_55_-PP yielded a significantly higher apparent K_d_ = 15.3 µM (95% CI: 9.5 to 32.8 µM) than the K_d_ obtained for UptA:C_55_-P (K_d_ = 5.7 ± 0.7 µM). Being within an order of magnitude, these dissociation constants suggest that UptA forms extensive contact with both forms of the lipid carriers but that the protein interacts more favorably with C_55_-P than C_55_-PP. We therefore conclude that UptA is more selective toward C_55_-P than C_55_-PP, thus confirming its preferred endogenous substrate.

### C_55_-P Binding to UptA Is Sensitive to pH.

UptA is a member of the DedA family of transporters whose cellular functions are potentially driven by proton-motive force and provide conditional fitness to bacteria under alkaline conditions (25, 26, 36). Membrane flippases such as MurJ, which is driven by proton-motive force (37), exhibit pH dependency in ligand binding (14). We therefore considered the possibility that substrate interactions of UptA might exhibit pH dependency. To this end, we equilibrated C_55_-P with UptA at a protein/ligand molar ratio of 1:4 in a buffer of the same composition but at pH 8.0 and 5.0. The resulting spectrum at pH 8.0 exhibits peaks consistent with monomeric UptA in an apo form, and in complex with 1 and 2 molecules of C_55_-P (*SI Appendix*, Fig. S4). We also observed UptA dimer in complex with up to two C_55_-P molecules, and the relative intensities of C_55_-P bound to the dimer is higher than to the monomer (*SI Appendix*, Fig. S4). This suggested that dimer interacts more favorably with C_55_-P than the monomer UptA. However, the peaks assigned to ligand-bound UptA are more intense at pH 8.0 compared to pH 5.0 by a factor of 3. These results imply that more favorable protein–ligand interactions occur under physiologically relevant pH conditions. As a negative control, we tested for pH sensitivity of C_55_-P binding to MraY, the latter being the downstream enzyme in the peptidoglycan pathway that couples UM5 to C_55_-P. To this end, we equilibrated MraY and C_55_-P at a molar ratio of 1:4 in a buffer of pH 8.0 and pH 5.0. In this case, the spectra show no significant difference in the intensity of C_55_-P bound to MraY at pH 8.0 and at pH 5.0 (*SI Appendix*, Fig. S4). We therefore conclude that environmental pH modulates the interaction of C_55_-P with UptA and potentially its transport mechanism.

### Amino Acid Residues Mediating C_55_-P Interactions with UptA.

**Fig. 3.**
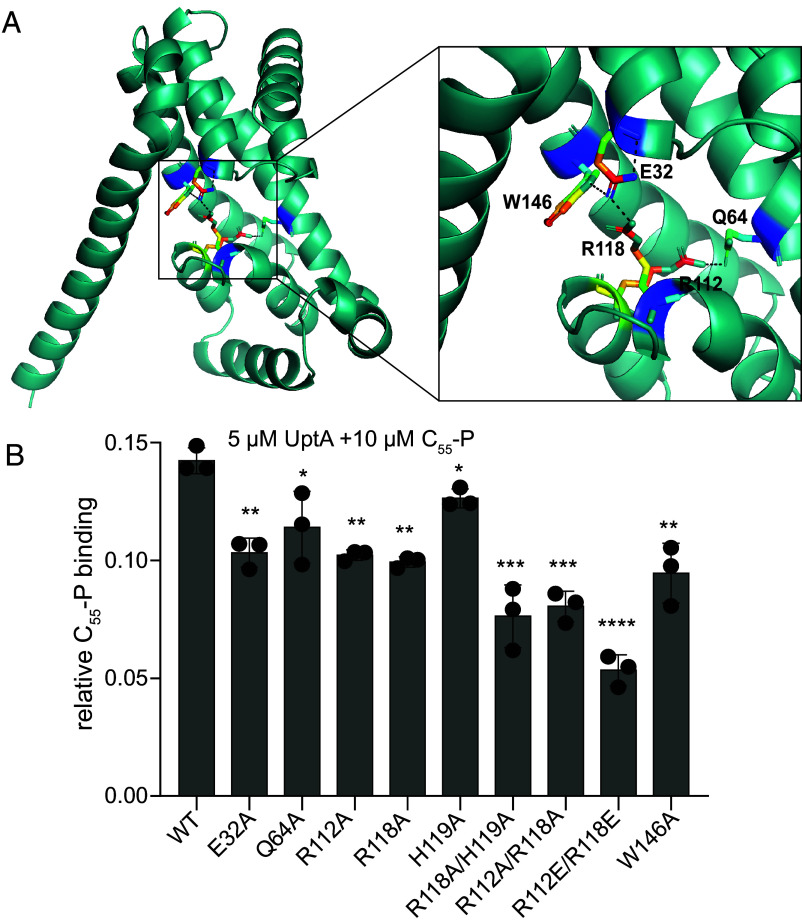
Probing the amino acid residues involved in UptA-C_55_-P binding. (*A*) Model UptA structure predicted by AlphaFold (AFO31823-F1) (22) and a zoomed view showing hydrogen bonding networks surrounding R118 and R112. R112–Q64: 3.1 Å; R118–E32: 2.7 Å; and E32–W146: 3.5 Å. Hydrogen bonding distances were computed using ProteinTools (38) and the image was processed in PyMOL. (*B*) Relative intensity of C_55_-P bound to UptA wild type and mutants. Bars represent the mean of three biological replicates with each data point shown in black, and error bars are SD. The double mutant at residues R112 and R118 caused the most significant reduction in C_55_-P binding. *P = 0.02 to 0.04, **P = 0.004 to 0.0012, ****P = 0.0002, ****P < 0.0001 from unpaired two-tailed *t* test by comparing mutants with wild type, n = 3.

Conserved arginine residues in the putative membrane reentrant loops of *E. coli* DedA proteins YqjA (R130) and YghB (R136) are important for the cellular role of these proteins (36). The corresponding arginine residues (R112 and R118) in *B. subtilis* UptA have been suggested to impair the cellular function of UptA by making the cells more susceptible to inhibition by amphomycin (23). How these residues impact substrate interactions, however, is not known. Based on the model structure of UptA predicted by AlphaFold, the R112 residue should engage Q64 while R118 is able to form hydrogen bonds with E32, H119, and W146 to potentially stabilize the membrane reentrant loops (Fig. 3*A*). To test for the impact of these residues on C_55_-P binding, we generated the E32A, Q64A, R112A, R118A, H119A, and W146A UptA single mutants. After purification of the proteins, using the same protocol as the wild type (WT) (*Materials and Methods* and *SI Appendix*, Fig. S5), we equilibrated aliquots of each protein variant (5 µM) with C_55_-P (10 µM) and recorded spectra using the same instrument settings. The resulting spectra exhibited peaks consistent with C_55_-P binding in all cases; but only a modest reduction in C_55_-P binding intensity was observed in the case of the Q64A and H119A mutants compared to the wild type (*SI Appendix*, Fig. S5 and Fig. 3*B*). Compared to the wild type UptA, we observed more significant reduction (27 to 30%) in C_55_-P binding with the mutants E32A, W146A, R112A, and R118A that participate directly in the predicted hydrogen bonding network (Fig. 3 *A* and *B*), suggesting that these residues are important for C_55_-P binding. We probe this observation further by investigating the double mutants R112A/R118A and R118A/R119A where the C_55_-P binding reduced more significantly (43% and 46%, respectively) than in the single mutants. We additionally generated a double mutant R112E/R118E in which the polarity of both native arginine residues in the wild type are reversed. We find that the R112E/R118E UptA exhibited 63% reduction in the intensity of C_55_-P binding compared to the wild type (Fig. 3*B*). These observations suggest that impaired hydrogen bonding between R118 and Q64 on the one hand and between R118 and E32 on the other hand could have also disrupted the C_55_-P binding sites, highlighting the roles of R112 and R118 in the binding of C_55_-P to UptA.

Since wild-type UptA displayed monomer–dimer equilibria and the dimer bind to C_55_-P more intensely than the monomer (cf. *SI Appendix*, Fig. S4), we probed this observation further using the UptA single amino acid mutant E32A since the latter consistently retained a higher dimer population than the wild type (*SI Appendix*, Fig. S6). We prepared and analyzed solutions containing 2.5 µM E32A UptA and 10 µM C_55_-P. The resulting spectra exhibited peaks assigned to UptA monomers and dimers in complex with C_55_-P (*SI Appendix*, Fig. S6). We find that the relative intensity of ligand-bound dimers is significantly higher than the ligand-bound monomers, and the ligand binding has only a modest impact on the monomer–dimer ratios. Together these results suggest that the ligand C_55_-P binds to dimeric UptA with a higher affinity than to the monomeric form.

### UptA Interacts More Favorably with C_55_-P than Its Shorter-Chain Analogs.

Next, we investigated the chain length selectivity of UptA by testing its interaction with geranylgeranyl phosphate (C_20_-P), hexaprenyl phosphate (C_30_-P), and C_55_-P; the latter representing the native form of the lipid carrier in the model organisms *B. subtilis* and *E. coli* (39). For this experiment, we incubated 4 µM UptA with a 10-fold molar excess of C_20_-P, C_30_-P, and C_55_-P at pH 8.0 and recorded spectra under the same conditions. For the UptA/C_20_-P sample, the spectrum exhibits low-intensity peaks corresponding by mass to UptA:C_20_-P complexes, leaving most of the protein in the apo form (*SI Appendix*, Fig. S7). In the case of UptA/C_30_-P, we observed more intense protein/ligand complexes, indicating a higher binding affinity of UptA to C_30_-P compared to C_20_-P (*SI Appendix*, Fig. S7). The spectra recorded for the UptA/C_55_-P mixture present peaks with the highest intensity of protein/ligand complexes (*SI Appendix*, Fig. S7), indicating a more favorable interaction for C_55_-P compared to the lipidic substrate with shorter aliphatic chains. The length and stereochemistry of lipid tails can influence their interactions with membrane proteins (40). Our data show that the lipid carrier with longer hydrophobic tails binds more favorably to UptA than the shorter ones, supporting our hypothesis that C_55_-P can form extensive contact with UptA to favor its binding interactions. Accordingly, the relative abundance of UptA in complex with C_55_-P is higher than in the case of UptA-bound C_30_-P and C_20_-P (*SI Appendix*, Fig. S7). Together, these data are consistent with C_55_-P being the preferred endogenous substrate for UptA.

### UptA Binds More Favorably to Phosphatidylglycerols than Phosphatidylethanolamines.

**Fig. 4.**
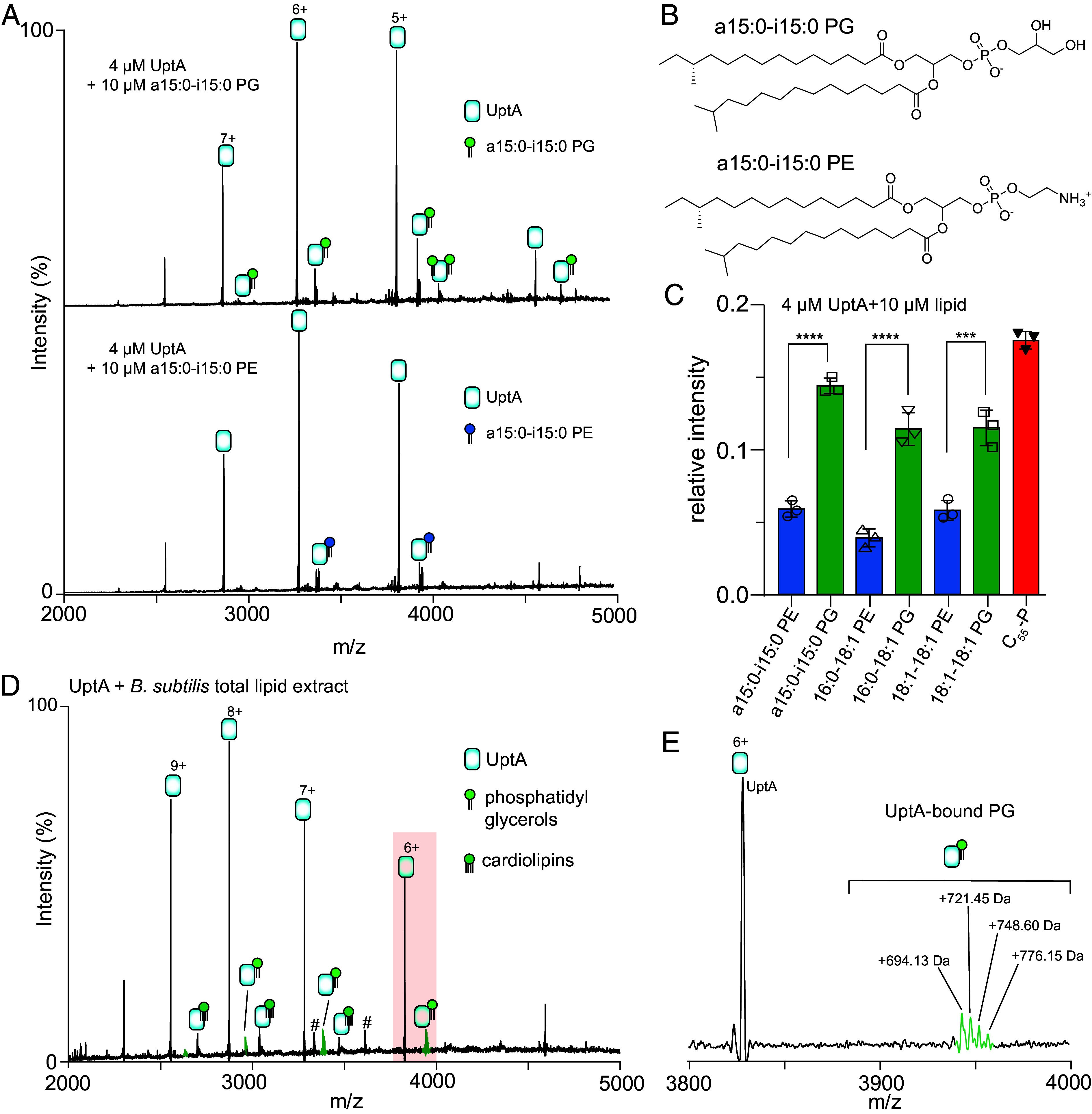
UptA binds phosphatidylglycerols. (*A*) Native mass spectrum for UptA equilibrated with a15:0-i15:0 PE and a15:0-i15:0 PG. UptA interacts more favorably with PG than PE. (*B*) Chemical structure of a15:0-i15:0 PE and a15:0-i15:0 PG. (*C*) Relative intensity of UptA bound to phospholipids and C_55_-P. UptA interacts more favorably with C_55_-P than with the phospholipids. The bar represents the mean of three independent replicates shown as data points and the error bars are SD. *P*-values are from two-tailed *t* tests with n = 3, ***P = 0.0003 to 0.0005, ****P < 0.0001. (*D*) Native mass spectrum of 4 µM UptA equilibrated with ~0.1 mg/mL total lipid extracts of *B. subtilis.* Peaks labelled # is a 43.3 kDa contaminant. (*E*) Zoomed view of a change state (6+), showing UptA adducts with phosphatidylglycerols.

Proteins belonging to the DedA family are a widespread family of transporters and are proposed to flip diverse lipids, including carrier lipids and membrane phospholipids. We therefore probed the interaction of UptA with membrane phospholipids to understand whether the latter could modulate its function. For this purpose, we selected anionic phosphatidylglycerols (PG) and zwitterionic phosphatidylethanolamines (PE), representing the major classes of phospholipid headgroups found in *B. subtilis* (41, 42). We recorded mass spectra of solutions containing 4 µM UptA incubated with 10 µM a15:0-i15:0 PE and 10 µM a15:0-i15:0 PG (a15:0, anteiso-pentadecanoic acid; i15:0, iso-pentadecanoic acid). The fatty acid composition of these lipids closely mimics those of native *B. subtilis* lipids (see below). The resulting spectra display peaks corresponding to UptA in apo and lipid-bound forms in both cases (Fig. 4 *A* and *B*). We observed the formation of 1:1 and 1:2 protein-lipid complexes of UptA with a15:0-i15:0 PG but only a 1:1 complex in the case of a15:0-i15:0 PE. Accordingly, the mean relative intensity of UptA in complex with a15:0-i15:0 PG is higher than for a15:0-i15:0 PE (Fig. 4*C*). We therefore hypothesize that UptA interacts more favorably with PG than with PE. We tested additional phospholipids (PG and PE) with comparable acyl chains. We find that 16:0-18:1 PG and 18:1-18:1 PG bind to UptA more intensely than 16:0-18:1 PE and 18:1-18:1 PE, respectively (Fig. 4*C*), supporting the hypothesis. Importantly, UptA binds to C_55_-P more intensely than any of these diacylglycerol phospholipids (Fig. 4*C*), in line with C_55_-P being its native substrate.

To verify the lipid binding preference of UptA with more native-like lipids, we prepared lipid extracts from *B. subtilis* membranes (*Materials and Methods*). We then incubate an aliquot of the resulting crude lipids (*SI Appendix*, Fig. S8) with UptA. The spectra yielded peaks assigned to UptA in complex with cardiolipins (~1,326 Da) and a range of phospholipids, predominantly 694 Da, 721 Da, 748 Da, and 776 Da species (Fig. 4 *D* and *E*). To identify these lipids, we performed tandem MS/MS, isolating the lipid species and subjecting it to collisional activation. The resulting spectra exhibited fragments at 152.99 Da (*SI Appendix*, Fig. S9), a diagnostic feature of the phosphatidylglycerol headgroup (43). The fatty acid fragments are mixed populations, predominantly 15:0-15:0, 15:0-17:0, 16:0-16:0, and 16:0-18:1 (*SI Appendix*, Fig. S9) which are the typical fatty acids from *B. subtilis* membrane phospholipids (42). We therefore conclude that UptA interacts more favorably with the anionic cardiolipins and phosphatidylglycerols than with the zwitterionic phosphatidylethanolamines in the membrane lipid extracts.

### C_55_-P Outcompetes Phospholipids for UptA Binding.

To investigate whether or not C_55_-P can outcompete phospholipids in binding to UptA, we incubated solutions containing UptA-DOPG with increasing concentrations of C_55_-P. Spectra were recorded after aliquots of the solution were equilibrated on ice for 15 min. The spectrum of UptA/DOPG mixture in the absence of C_55_-P reflected the binding of 1 to 2 molecules of DOPG per UptA (*SI Appendix*, Fig. S10). The presence of C_55_-P in the sample yielded a new peak corresponding to UptA:C_55_-P complex and simultaneously caused attenuation of the intensity DOPG-bound UptA in a concentration-dependent manner. At the highest C_55_-P concentration tested (20 µM), C_55_-P has displaced most of DOPG from UptA (*SI Appendix*, Fig. S10). We therefore hypothesised that phospholipids should be unable to outcompete C_55_-P bound to UptA. To test this hypothesis, we incubated UptA with C_55_-P and then equilibrated aliquots with an increasing concentration of DOPG. Indeed, C_55_-P remains bound to UptA even in the presence of excess DOPG (20 µM) (*SI Appendix*, Fig. S10), indicating that phospholipids could not significantly displace C_55_-P from UptA. Analogous experiments with DOPE show that C_55_-P effectively outcompete DOPE for UptA binding but the latter could not efficiently displace C_55_-P from UptA (*SI Appendix*, Fig. S10). Together, this study shows that UptA interacts more favorably with C_55_-P than with membrane phospholipids, thereby establishing C_55_-P as the preferred endogenous substrate.

### Lipopeptide Antibiotics Disrupt UptA:C_55_-P Complex.

We next investigate how UptA and its complex with C_55_-P interact with a range of cell-wall targeting antibiotics including the lipopeptides daptomycin and amphomycin. Daptomycin is known to kill bacteria by depolarizing the membrane (44) and by complexing with lipid II (45). We investigated whether daptomycin affects C_55_-P binding to UptA by recording spectra for solutions containing 2.5 µM UptA, 10 µM C_55_-P, and different concentrations of daptomycin. In the absence of antibiotics, the spectrum exhibited peaks assigned to UptA in apo form, and in complex with 1 and 2 C_55_-P molecules (*SI Appendix*, Fig. S11). In the presence of daptomycin, we observed additional peaks including those corresponding to the binary complex UptA:daptomycin and the ternary complex UptA:C_55_-P:daptomycin (*SI Appendix*, Fig. S11). Further increases in daptomycin concentration resulted in higher intensity of these complexes, indicating that daptomycin interacts with UptA but it does not directly compete against C_55_-P binding to the protein.

**Fig. 5.**
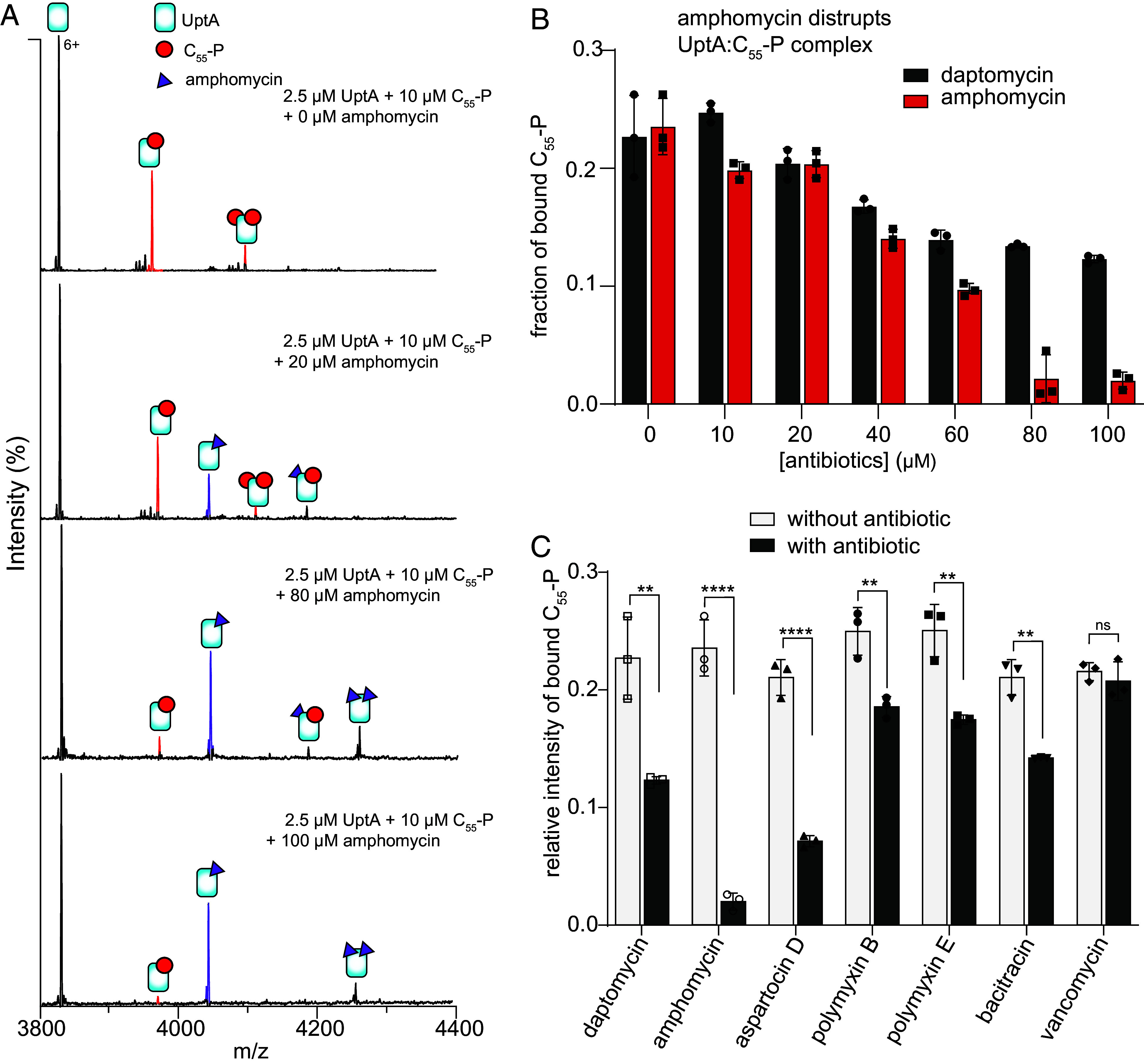
UptA function can be inhibited by lipopeptide antibiotics. (*A*) Mass spectra (6+ charge state) for solutions containing 2.5 µM UptA and 10 µM C_55_-P incubated with 0 to 100 µM amphomycin. (*B*) Relative intensity of C_55_-P bound to UptA in the presence of amphomycin and daptomycin. The bar represents the mean of three independent replicates shown as data points and error bars are SD. Amphomycin destabilizes the UptA:C_55_-P complex whereas daptomycin has a modest impact. (*C*) Fraction of C_55_-P bound to UptA in the presence of cell wall targeting antibiotics. Each sample contains 2.5 µM UptA, 10 µM C_55_-P and 100 µM of indicated antibiotic, and spectra were acquired using the same instrument settings and collisional activation of 75 V. The bar represents the mean of three independent replicates shown as data points, and the error bar represents SD. *P*-values from two-tailed *t* tests with n = 3; **P = 0.001 to 0.006; ****P < 0.0001, ns, not significant.

Amphomycin and derivatives are known to kill bacteria by forming complexes with free C_55_-P (46), however, whether or not this involves a direct interaction with a protein cellular target is not known. We therefore investigated how amphomycin affects the UptA:C_55_-P complex. We recorded spectra for solutions containing 2.5 µM UptA, 10 µM C_55_-P and then equilibrated aliquots with increasing concentrations of amphomycin. The spectra exhibited peaks consistent with amphomycin binding to UptA, in addition to the C_55_-P binding (Fig. 5*A*). We find that the peak intensities of the UptA:C_55_-P complex reduce with respect to an increase in the concentration of amphomycin (Fig. 5*B*). Compared to the case of daptomycin, little-to-no UptA:C_55_-P complexes remained in the presence of 10-fold molar excess of amphomycin (Fig. 5*B*), suggesting that amphomycin destabilize the ternary complex to sequester C_55_-P. We then performed similar experiments with aspartocin D, an analog of amphomycin. The resulting spectrum shows that aspartocin D similarly binds to UptA and also induces dissociation of UptA:C_55_-P complex (Fig. 5*C*).

The amphiphilic nature of daptomycin and amphomycin raises the possibility that these lipopeptide antibiotics may promiscuously bind to UptA in a nonspecific manner. We probe this possibility by recording spectra for UptA:C_55_-P solutions in the presence of 100 µM polymyxin B and 100 µM polymyxin E. These polymyxins are membrane-disrupting lipopeptides that target the outer membrane lipopolysaccharides in Gram-negative bacteria (47) but without a known affinity for C_55_-P or UptA. We find that, unlike daptomycin and amphomycins, the polymyxins do not bind to UptA (*SI Appendix*, Fig. S11), indicating that not every lipopeptide can bind to UptA. The ability of daptomycin and amphomycin analogs to directly interact with UptA therefore suggests that these lipopeptides may render a fraction of the cellular pool of UptA inaccessible to C_55_-P. Notably, polymyxin B and polymyxin E similarly caused attenuation of C_55_-P binding to UptA (*SI Appendix*, Fig. S10), but this effect is less significant compared to the impact of amphomycin analogs (Fig. 5*C*). Polymyxins are not known to form a stable complex with C_55_-P, however, their cationic nature suggests the possibility of electrostatic interaction with C_55-_P molecules, similar to their affinity for the negatively charged lipopolysaccharides (48).

To verify these results, we studied the impact of bacitracin and vancomycin on UptA:C_55_-P interactions. Bacitracin and vancomycin are peptide antibiotics whose mode of action involves sequestration of C_55_-PP and lipid II, respectively (49, 50). To this end, we prepared UptA:C_55_-P mixtures and then equilibrated aliquots with bacitracin and vancomycin. We find that bacitracin and vancomycin bind to UptA but with very low intensities (*SI Appendix*, Fig. S11), indicating weak affinity interactions. In line with the specificity of bacitracin for C_55_-PP rather than C_55_-P, this antibiotic modestly competes against C_55_-P binding to UptA (*SI Appendix*, Fig. S11 and Fig. 5*C*). By contrast, vancomycin has no observable impact on C_55_-P binding (*SI Appendix*, Fig. S11 and Fig. 5*C*). Thus, the nonlipopeptide antibiotics exhibit weak affinity for UptA and had only a minor impact on UptA:C_55_-P interactions. Taken together, our results highlight that the lipopeptides such as amphomycin and aspartocin D induce dissociation of UptA:C_55_-P complexes and may therefore interfere with peptidoglycan pathway by binding directly to UptA in addition to their known propensity to form complexes with free C_55_-P molecules (46).

## Discussions

**Fig. 6.**
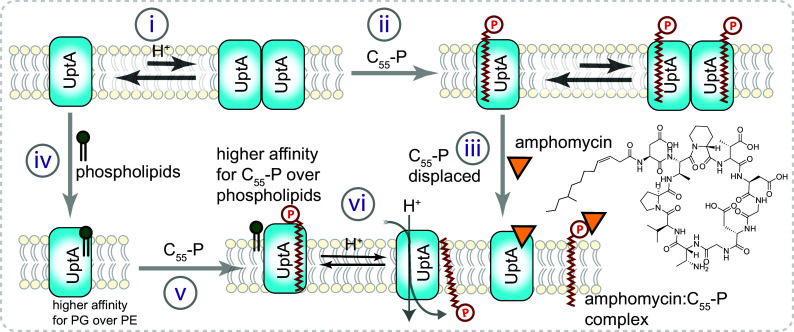
Insights from native MS into the interaction of UptA with lipidic substrates and antibiotics. (i) UptA exhibits monomer–dimer equilibria, with more dimer at pH 8.0 than pH 5.0. (ii) UptA interacts with C_55_-P more favorably than C_55_-PP, C_30_-P, and C_20_-P. (iii) Amphomycin destabilizes the UptA:C_55_-P complex and sequesters C_55_-P, thus effectively out-competing the flippase for its carrier lipid substrate. (iv) UptA binds phospholipids, with a higher preference for the anionic than zwitterionic phospholipids. (v) UptA interacts with C_55_-P more favorably than phospholipids, highlighting its preferred lipid substrate. (iv) Based on the proton dependency of other DedA proteins (53) and the pH dependency of UptA–C_55_-P interactions, we propose that the binding of H^+^ to UptA enables translocation of C_55_-P and its subsequent release for the next round of PG precursor synthesis.

In this study, using native MS, we investigated the interplay of multiple lipidic substrates and antibiotics with UptA (summarized in Fig. 6). Specifically, we report that purified UptA exists as an equilibrium of monomers and dimers, suggesting that DedA proteins might form dimers in the native membrane when lateral pressures in the lipid bilayers (51) might prevent their dissociation. In line with the dependency of DedA family proteins on proton motive force (52), we observed less C_55_-P binding to UptA in the presence of excess H^+^ and that the monomer–dimer ratio of UptA is sensitive to pH rather than to C_55_-P binding. This feature contrasts with those of the upstream peptidoglycan enzymes MraY (35, 53) and UppP (54, 55) whose dimerization is promoted by lipid binding.

Our studies also show that UptA selectively binds to C_55_-P over C_55_-PP and that the protein interacts more favorably with C_55_-P than the shorter chain analogs C_30_-P and C_20_-P. This implies that the hydrophobic tail of C_55_-P makes extensive contact with UptA, promoting the binding interactions and potentially facilitating the transport of carrier lipids. The higher affinity of UptA toward the monophosphate form of the carrier lipid, rather than to the diphosphate form, favors a mechanism in which UptA mediates the transport of C_55_-P rather than both C_55_-P and C_55_-PP. We further show that UptA interacts more favorably with the anionic lipids than the zwitterionic lipids and that these lipids are readily displaced by C_55_-P. This binding preference agrees with UptA being responsible for C_55_-P rather than the phospholipid translocation (23). Competition between anionic lipids, which are more abundant in the native lipid bilayer than C_55_-P, suggests that both lipids bind near the same sites on UptA and that the local lipid environment of UptA can potentially modulate its cellular function.

We provided molecular-level evidence that the conserved arginine (R112 and R118) residues in the putative membrane reentrant helices of UptA are important for its substrate binding interactions, confirming earlier reports that *B. subtilis* strains bearing these mutations on UptA are more susceptible to inhibition by MX2401, a derivative of amphomycin (23). Despite the combined cellular pool of C_55_-PP and C_55_-P being limited to ~1.5 × 10^5^ molecules per cell (56), they mediate the transport of glycan-bearing components of the cell wall peptidoglycan, teichoic acid, and capsular polysaccharide (57). Therefore, antibiotics that block C_55_-P and its flippases are potential drug targets that can potentially inhibit multiple pathways and thus mitigate drug resistance. Accordingly, bacitracin and teixobactin form complex with C_55_-PP (58, 59) while amphomycin analogs inhibit bacterial cell wall formation by forming complexes with C_55_-P (23, 46, 60, 61). Herein we show that amphomycin and aspartocin D can additionally bind directly to UptA and induce dissociation of the UptA:C_55_-P complex, a finding that may guide the development of new antibiotic analogs to inhibit the flippase function of UptA and its homologs.

## Materials and Methods

Detailed experimental procedures are provided in *SI Appendix*. Briefly, UptA (wild type and mutants) was expressed in *E. coli* C43(DE3) cells (Lucigen) with GFP fusion on the C-terminus and solubilized from the membrane fraction by *n*-dodecyl-*β*-*D*-maltopyranoside (DDM). After purification by affinity chromatography, the GFP was cleaved by TEV protease and removed, and UptA was further purified by size-exclusion chromatography in a buffer containing 20 mM Tris-HCl (pH 8.0), 200 mM NaCl, 0.02% DDM. Before measurements, proteins were buffer-exchanged into 0.05% LDAO, 200 mM ammonium acetate at the desired pH using a centrifugal buffer exchange device (Micro Bio-Spin 6, Bio-Rad). Stock solutions of lipids were prepared from chloroform/methanol solution by evaporating aliquots of known volume in a SpeedVac. After drying, the lipid films were weighed and resuspended to a final concentration of 0.5 mM in 200 mM ammonium acetate and 0.05% LDAO by vertexing. Stock solutions of 0.5 mM amphomycin, aspartocin D, daptomycin, polymyxin B, polymyxin E, and bacitracin were made in the same buffer. All experiments were repeated three times from newly prepared stock solutions. Native mass spectrometry was performed on a Q-Exactive hybrid quadrupole-Orbitrap mass spectrometer (Thermo Fisher Scientific, Bremen, Germany), and lipid identification was performed on an Orbitrap Eclipse Tribrid mass spectrometer (Thermo).

## Supplementary Material

Appendix 01 (PDF)

## Data Availability

Mass Spectrometry .raw files supporting the findings of this study have been deposited in the Figshare database at https://doi.org/10.6084/m9.figshare.27003172 (62).
