# Supplementary material for: Lipopeptide antibiotics disrupt interactions of undecaprenyl phosphate with UptA
Source: Proc Natl Acad Sci U S A. 2024 Oct 3;121(41):e2408315121. doi: 10.1073/pnas.2408315121 (PMC11474028; doi:10.1073/pnas.2408315121)
Supplement: Supplementary file 1 — Appendix 01 (PDF) [file pnas.2408315121.sapp.pdf]

PNAS

Supporting Information for Lipopeptide antibiotics disrupt interactions of undecaprenyl phosphate with UptA

Abraham O. Oluwole[1,2*], Neha V. Kalmankar[1,2], Michela Guida[2,3], Jack L. Bennett[1,2], Giovanna Poce[3], Jani R. Bolla[2,4] Carol V. Robinson[1,2*]

[1]Department of Chemistry, University of Oxford, South Parks Road, Oxford, OX1 3QZ, UK. [2]The Kavli Institute for Nanoscience Discovery, University of Oxford, Oxford, South Parks Road, Oxford, OX1 3QU, UK. [3]Department of Chemistry and Technologies of Drug, Sapienza University of Rome, 00185 Rome, Italy. [4]Department of Biology, University of Oxford, South Parks Road, Oxford, OX1 3RB, UK.

*Email: abraham.oluwole@chem.ox.ac.uk; carol.robinson@chem.ox.ac.uk

This PDF file includes:

Methods
Figures S1 to S11
Table S1
SI References

Methods

Chemicals

C_{55}-PP, C_{55}-P, C_{30}-P and C_{20}-P were purchased from Larodan. Phospholipids – a15:0-i15:0 PE, a15:0-i15:0 PG, 16:0-18:1 PE, 16:0-18:1 PG, 18:1-18:1 PE, 18:1-18:1 PG – were from Avanti Polar Lipids. Amphomycin and aspartocin D were purchased from Cayman; while Daptomycin and bacitracin were purchased from Merck. *n*-dodecyl-*β*-D-maltoside (DDM), *n*-decyl-*β*-D-maltoside (DDM), n-octylglucoside (OG) and *n*-dodecyl-*N,N*-dimethylamine-*N*-oxide (LDAO) were from Anatrace.

Plasmids

A codon-optimised version of *Bacillus subtilis* UptA (yngC) (Integrated DNA Technologies) was inserted into a modified pET15b vector between NdeI and NheI endonuclease restriction sites by In-Fusion HD cloning kit (Takara Bio). The resulting plasmid encodes UptA fused to GFP-His_6 on the C-terminus via a TEV-protease cleavable site. Plasmids for UptA mutants were generated by site-directed mutagenesis with CloneAmp HiFi PCR Premix (Takara Bio) using pairs of oligonucleotides listed in Table S1. The plasmid pET26-MBP-MraYaa bearing HRV3C protease cleavage site between the MBP and the wild-type MraY was a kind gift from S-Y Lee (Addgene plasmid #100166) (1).

Table S1: Oligonucleotides used to generate plasmids for the expression of UptA mutants.

Primers	Sequence
E32AF	TCCAAGCGCAATTGTATTGGCCTACGGTGG
E32AR	ACAATTGCGCTTGGAATAATTTCAATCATCAGT
Q64AF	GATCGCCGCAATTTTCATTTACTGGATTGGCCGT
Q64AR	AAAATTGCGGCGATCGTTCCCCCGATA
R112AF	TTCTGCTGCCTTCATCCCTGTAGTCCGTCATGC
R112AR	ATGAAGGCAGCAGAAAACACAACCCCCG
R118AF	TGTAGTCGCACATGCTATTTCGATCCCAGCCG
W146AF	CATTCCTGCCTCCATCCTGTTCGTTTACCTGG
W146AR	ATGGAGGCAGGAATGATGGCAAGTACTGTC
R112A/R118A_Fwd	TTCTGCTGCCTTCATCCCTGTAGTCGCACATGCTATTTCGATCCCA GCCG
R112A/R118A_Rev	GCATGTGCGACTACAGGGATGAAGGCAGCAGAAAACACAACCCC CG
R112E/R118E_Fwd	ATTCATCCCTGTAGTCGAACATGCTATTTCGATCCCAGCCG
R112E/R118E_Rev	ACTACAGGGATGAATTCAGCAGAAAACACAACCCCCG

Expression and purification of UptA

Plasmids were used to transform chemically competent *E. coli* C43(DE3) cells (Lucigen) and selected on LB/Agar containing 100 µg/ml ampicillin at 37 °C. Single colonies were used to inoculate 60 mL LB containing 100 µg/ml ampicillin and were grown at 37 °C overnight for 16 h. For large-scale protein expression, 10 mL of the overnight culture was aseptically transferred into 1 L of LB/ampicillin and cells were allowed to grow at 37°C until the $OD_{600\ nm}$ reached 0.6-0.8 after which the temperature was lowered to 18 ^{o}C. Expression of UptA-GFP was induced by adding isopropyl-β-D-thiogalactopyranoside (IPTG) at a final concentration of 0.5 mM UptA-GFP and shaking for 20 h at 18 °C. Cells were harvested by centrifugation at 5000 × *g* and lysed immediately or stored at -80 ° C until required.

Cells were thawed on ice and resuspended in a buffer containing 50 mM Tris-HCl (pH 7.4), 200 mM NaCl, 10 µg/mL deoxyribonuclease I (DNAse I), 5 mM $MgCl_2$, 5 mM β-mercaptoethanol and EDTA-free protease inhibitor cocktail tablets. The cells were then disrupted with a microfluidiser (Microfluidics) operated at 20,000 psi. Non-lysed cells and debris were pelleted by centrifugation at 20,000 × *g* for 20 min and the clarified lysate was retained. The membranes fraction was then pelleted by ultracentrifugation at 100,000 x g for 1 h. They were resuspended in a buffer containing 20 mM Tris-HCl (pH 8.0), 300 mM NaCl, 20% glycerol. Membrane aliquots were either solubilised immediately or flash-frozen in liquid nitrogen and stored at -80°C. The membrane was solubilised by incubation with DDM at a final concentration of 1% (w/v) for 60 min. Non-solubilised aggregates were removed by centrifugation at 20,000×g for 20 min and the supernatant was loaded onto a 5-ml HisTrap HP column preequilibrated in buffer A (20 mM Tris-HCl (pH 8.0), 200 mM NaCl, 10% glycerol, 0.03% DDM and 20 mM imidazole). After binding of the His-tagged protein, the column was washed with 100 mL of buffer A, and then with 50 mL of buffer B (20 mM Tris-HCl (pH 8.0), 200 mM NaCl, 10% glycerol, 0.03% DDM and 50 mM imidazole). UptA-GFP was eluted from the column with buffer C (20 mM Tris-HCl (pH 8.0), 200 mM NaCl, 10% glycerol, 0.02% DDM and 300 mM imidazole). To remove imidazole and to cleave the GFP fusion, eluted protein was mixed with TEV protease and dialysed against buffer D (20 mM Tris-HCl (pH 8.0), 200 mM NaCl, 10% glycerol, 0.02% DDM) at 4 ^{o}C for 16 h.

The digestion mixture was subsequently incubated with 2-mL bed volume of nickel nitriloacetic acid (NTA) agarose resin to remove His-tagged GFP and TEV protease. Tag-free UptA was collected as the flowthrough, concentrated using an Amicon centrifugal concentrator with nominal molecular weight cut-off 50 kDa. The protein was finally purified by size-exclusion chromatography (SEC) using a 24-mL Superdex 200 Increase column. The SEC buffer was 20 mM Tris-HCl (pH 8.0), 200 mM NaCl, 0.02% DDM. UptA was concentrated to ~200 uM, aliquoted, flash-frozen flash in liquid nitrogen, and stored at -80 °C until use. Concentration was measured using a Biomate UV spectrophotometer at 280 nm using extinction coefficients calculated from their predicted amino acid sequences. UptA mutants were purified in 3 biological replicates, using the same procedures as for the wild type.

Expression and purification of MraY.

MraY from *Aquifex aeolicus* was purified as previously described (3). Briefly, the plasmid pET26-HisMBP-MraYaa was used to transform chemically competent *E. coli* C41(DE3) cells (Lucigen) and selected on LB/Agar containing 50 µg/ml kanamycin at 37 °C. Cells were grown in Terrific Broth media supplemented with 50 µg/ml kanamycin at 37°C until the $OD_{600\ nm}$ reached 0.8. Protein expression was induced by adding IPTG at a final concentration of 1 mM for 4 h at 37 ^{o}C. Cells were harvested by centrifugation at 5000 × *g* and lysed and solubilized by incubation with 1% DDM for 1 h. His-tagged MBP-MraY was purified by affinity chromatography and digested by HRV3C protease. MraY was isolated by SEC into a buffer containing 20 mM Tris-HCl (pH 8.0), 200 mM NaCl, 0.02% DDM.

Denaturing SDS-PAGE

Denaturing SDS-PAGE was performed using a precast NuPAGE™ Novex 12% Bis-Tris Mini protein gel (Invitrogen) according to the manufacturer's specifications. 30 µL of 0.3 µg/µL protein were mixed with 10 µL of 4X LDS sample loading buffer (Invitrogen) and heated at 80 °C for 5 min. 20 µL of the resulting samples were then loaded into in each well. 5 µL PageRuler™ prestained protein ladder (Thermo Scientific) was loaded into a well as molecular weight marker.

Electrophoresis was performed at room temperature for 36 min using a constant voltage (200 V) in 1X solution of NuPAGE™ MES SDS running buffer (Invitrogen). The gel was stained with Quick Coomassie stain solution (Neo Biotech) for 60 min and rinsed with water for 24 h before imaging on iBright™ FL1500 imaging system (Thermo).

Lipid extracts from *B. Subtilis*

B. subtilis strain 168 (ATCC 23857) was cultivated in 200 mM LB media at 37 ^{o}C until $OD_{600\ nm}$ 1.2 under aurobic conditions. Cells were harvested by centrifugation at 4,000 *g* for 20 minutes and resuspended in 20 mM Tris-HCl (pH 8.0), 150 mM NaCl. Cells were lysed by 5-6 passes through a microfluidizer at 20,000 psi. The lysates were clarified by centrifugation at 4,000 *g* for 10 minutes, and the totoal membrane was pelleted at 120,000 *g* for 1 h. The membrane pellet was ressupended in 20% sucrose, 50 mM Tris-HCl (pH 8.0), and loaded as the topmost layer of a 4-layer sucrose gradient: 20% (4 mL), 36% (3 mL), 51% (8 mL) and 55% (2 mL) (4). After centrifugation at 120,000 *g* for 20 hrs, the cytosolic membrane was collected at the interface of 36% and 45% sucrose layers, and washed 3 times with 1 M ammonium acetate by dilution-centrigugation at 120,000 *g* for 30 min. The membrane pellet was homogenised in 2 mL of 1 M ammonicum acetate and stored in -80 ^{o}C until use. Lipids were extracted by homogenising 0.8 mL of membrane with 1 mL of 1:1 vol/vol) chloroform-methanol mixture in a glass vial followed by vortexing. To separate the aqueous and the organic pahses, adding 1 mL chloroform was added, and the mixture was centrifuged at 3,000 *g* for 5 min. The chloroform layer containing the total lipids was recovered into a clean glass vial, and the organic solvent was evaporated in a SpeedVac for 16 h. Lipid film was resuspended 200 mM ammonium acetate, 0.05% LDAO (pH 8.0) to ~0.5 mg/mL for the binding experiments.

Sample preparation for native MS

Before measurements, the protein was buffer-exchanged into 0.05% LDAO, 200 mM ammonium acetate ("MS buffer") at the desired pH using a centrifugal buffer exchange device (Micro Bio-Spin 6, Bio-Rad). Stock solutions of lipids were prepared from chloroform/methanol solution by evaporating aliquots of known volume in a SpeedVac. After drying, the lipid films were weighed and resuspended to a final concentration of 0.5 mM in 200 mM ammonium acetate (pH8) and 0.05% LDAO by vertexing. Stock solutions of 0.5 mM amphomycin, aspartocin D, daptomycin and bacitracin, were made in the same buffer. All experiments were repeated three times from newly prepared stock solutions.

Native mass spectrometry

About 3 μL protein aliquot was transferred into a gold-coated borosilicate capillary (Harvard Apparatus) and was mounted on the nano ESI source of a Q-Exactive hybrid quadrupole-Orbitrap mass spectrometer (Thermo Fisher Scientific, Bremen, Germany). The instrument settings were 1.2 kV capillary voltage, S-lens RF 200%, argon UHV pressure 3.3×10^{-10} mbar, the capillary temperature was set to 200 °C, and resolution of the instrument was set to 17,500 at a transient time of 64 ms. Voltages of the ion transfer optics –injection flatapole, inter-flatapole lens, bent flatapole, and transfer multipole were set to 5, 3, 2, and 30 V respectively. The noise level was set at 3. Unless otherwise stated, proteins were activated by applying 75 V in the high-energy collisional dissociation cell without in-source trapping. For the MS^2 spectra, parent ions were isolated using an in-source trapping voltage of -200 V, and then fragmented by applying 150-200 V in the high-energy collision-induced dissociation (HCD) cell. Lipids bound to UptA from the total lipid extracts were identified on an Orbitrap Eclipse Tribrid mass spectrometer (Thermo). Peaks corresponding to the parent ions in the negative ESI spectra were isolated within ±1.0 Th and then activated with HCD voltage 10-30%. To identify endogenous modifications using native top-down mass spectrometry, the 6+ charge state of monomeric UptA was isolated (3828 ± 5 Th) and activated using HCD (200 V) with reduced trapping pressures. The resulting fragments were detected in the Orbitrap (R = 280,000 @ *m*/*z* 200), averaging ~500 transients to maximise the signal-to-noise ratio. Theoretical fragments from N-terminally formylated UptA were matched to the MS^2 spectrum (3 ppm) using TDValidator (Proteinaceous) and manual validation. Data were visualised and exported for processing using the Qual browser of Xcalibur 4.1.31.9 (Thermo Scientific).

Spectral deconvolution was performed using UniDec 6.0.2 (2). Relative binding affinities were obtained from deconvoluted spectra by dividing the intensity of ligand-bound protein peaks by the sum of the intensities of ligand-bound and ligand-free protein peaks. The mean and standard deviation of these fractional binding intensities from three independent experiments were plotted against the concentrations of the ligand C_{55}-P or C_{55}-PP. To obtain the apparent dissociation constant K_d, the mean relative intensities at different concentrations were fitted globally using GraphPad Prism 10.2.0 to Eq. (1):

$$y = \frac{B_{max} x^h}{x^h + K_d{}^h} \quad (1)$$

Where y is the mean relative intensity and x is the corresponding ligand concentration. B_{max} is the maximum specific binding, K_d is the apparent dissociation constant, and h is the Hill coefficient.

Supplementary Figures

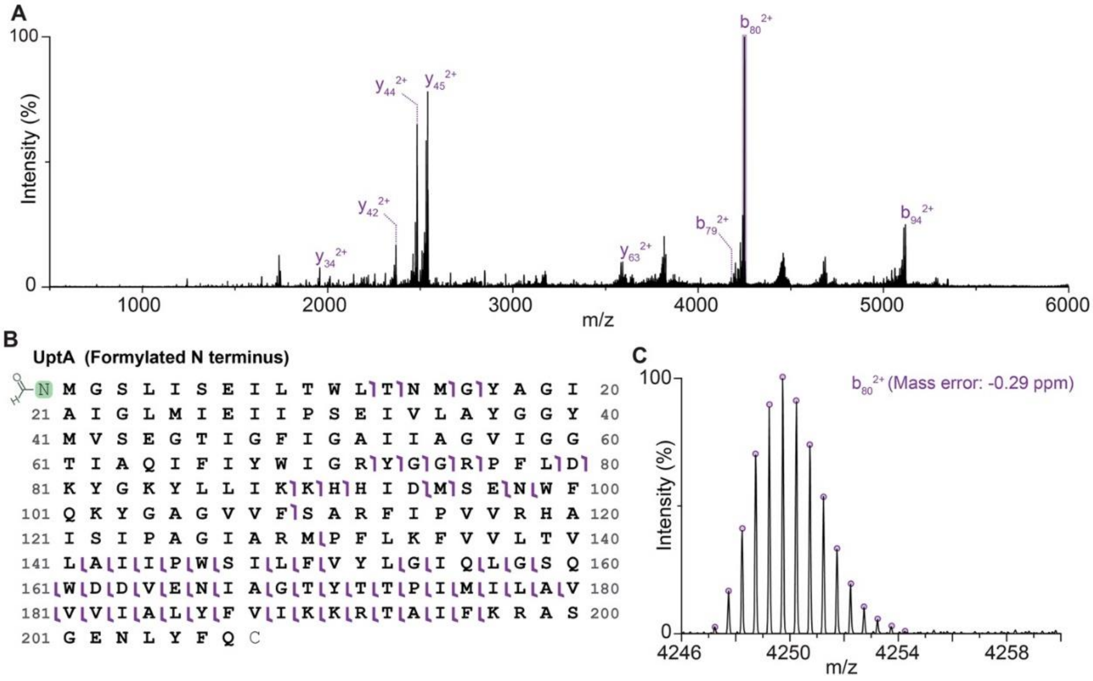

Fig. S1. UptA is formylated at the N-terminus. A. HCD MS/MS spectrum of UptA (6+). The eight most abundant assigned ions are labelled. **B**. Sequence map of the UptA construct used in this study. Terminal *b*- and *y*-type fragment ions observed in the MS/MS spectrum are indicated. **C.** Expanded view of the peak corresponding to b_{80}^{2+} ion**.** The theoretical isotopic envelope is overlaid.

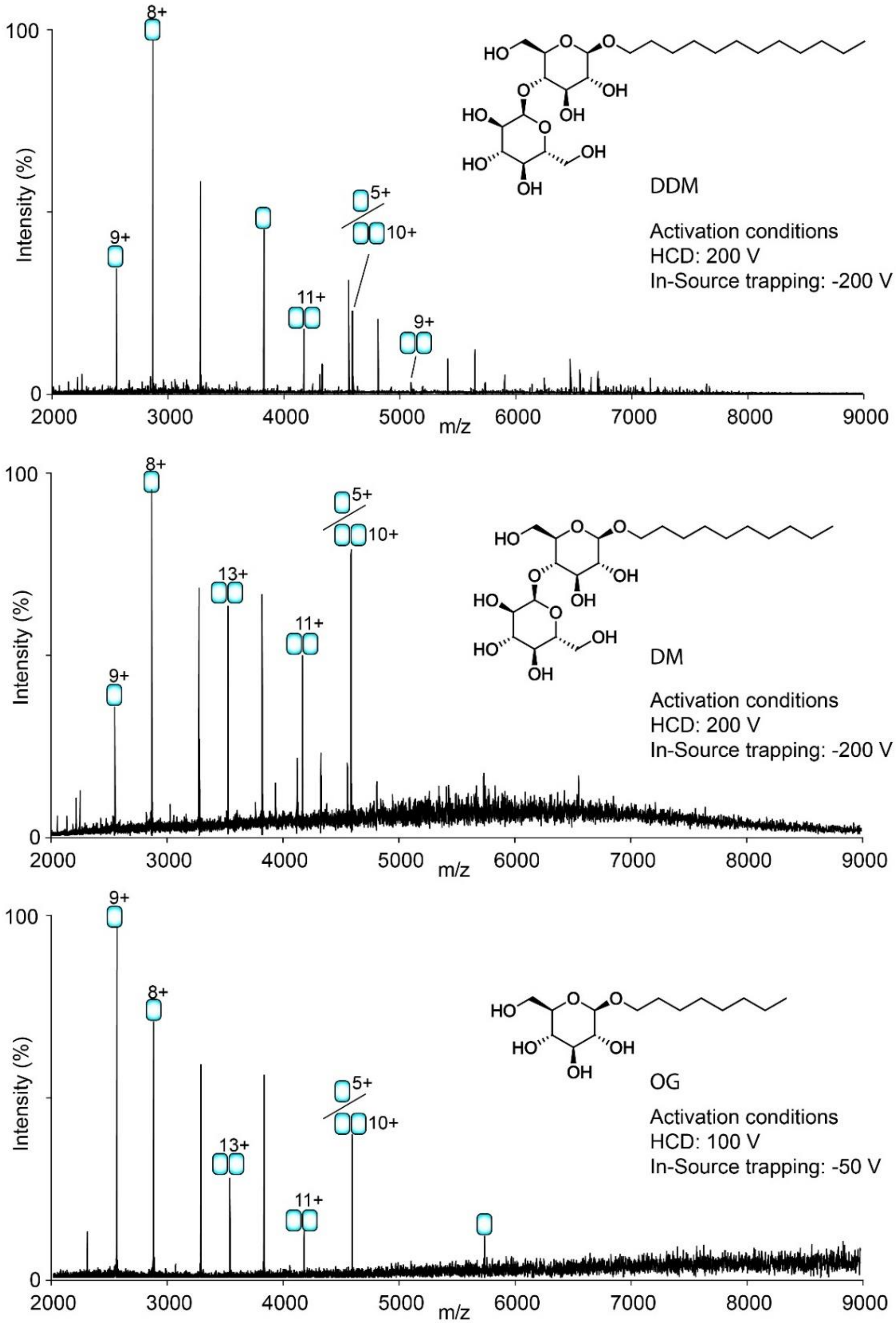

Fig. S2. Oligomeric state of UptA in different detergents. Shown are the spectra recorded for UptA in buffer containing 200 mM Ammonium acetate (pH 8.0) supplemented with indicated detergents. The instrument was tuned differently in each case to maximise the transmission of dimeric species.

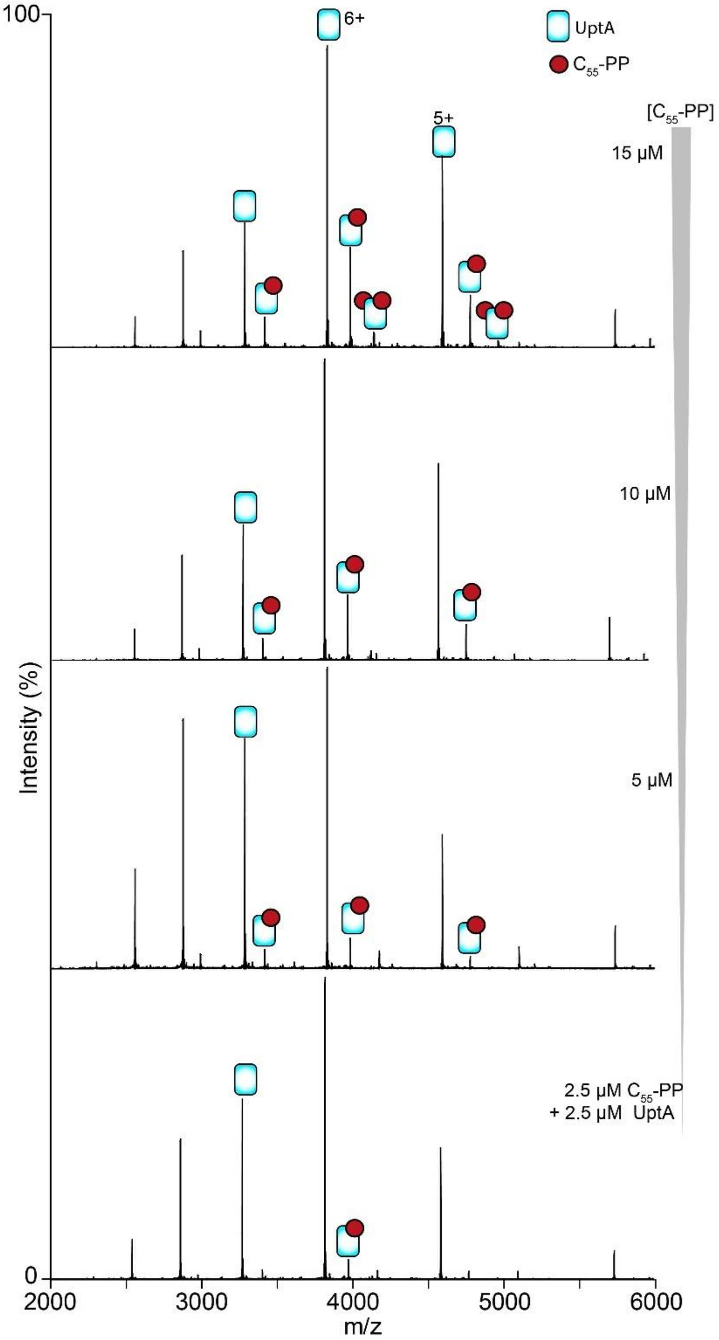

Fig. S3. UptA:C_{55}-PP binding interactions. Spectra for UptA incubated with different concentrations of C_{55}-PP in a buffer containing 200 mM ammonium acetate (pH 8), 0.05% LDAO. UptA bind C_{55}-PP but with lesser intensities compared to C_{55}-P (cf. Fig. 2A).

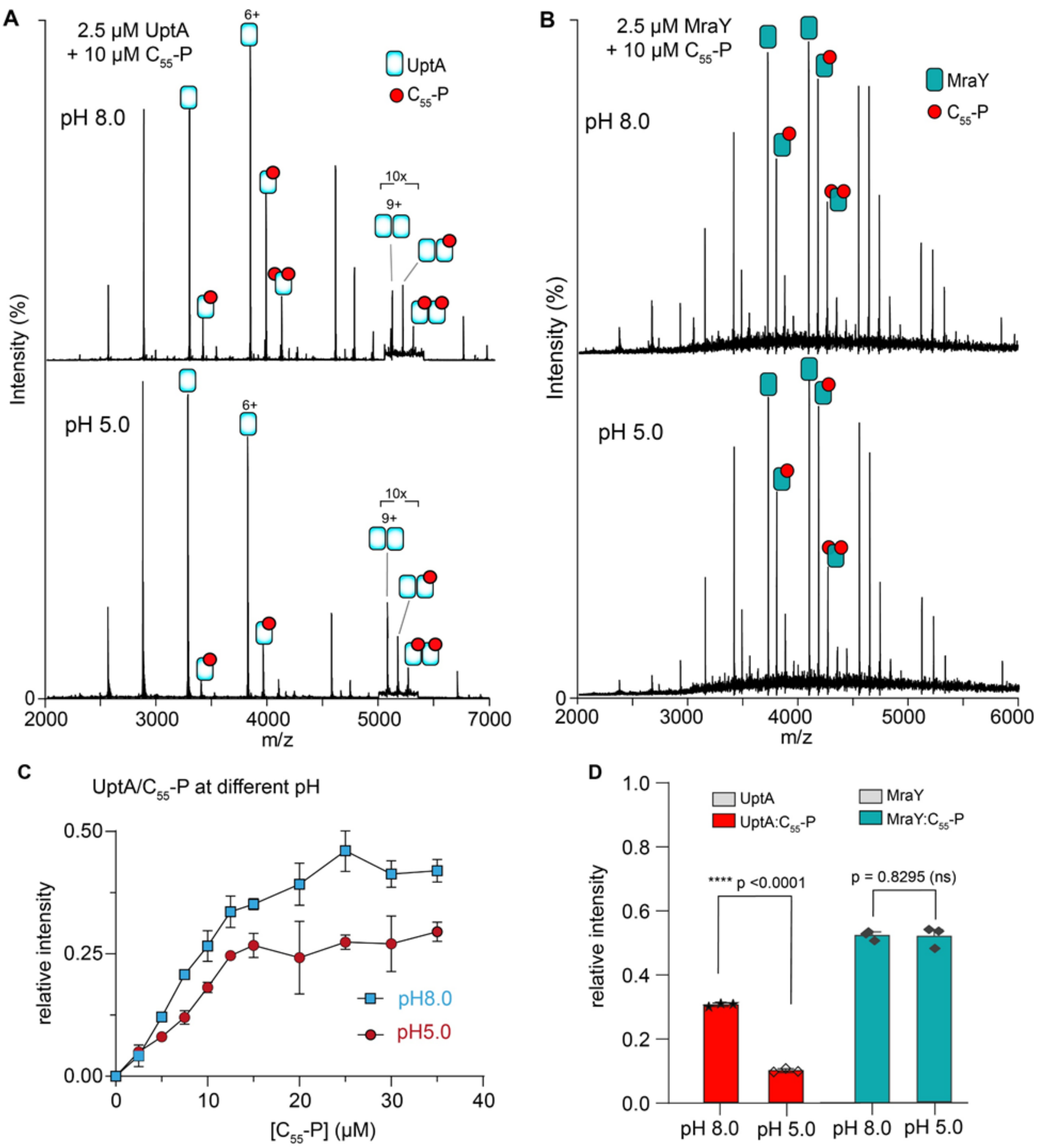

Fig. S4. pH dependency of C_{55}-P binding interactions with UptA and MraY. A,B. Spectra for UptA/C_{55}-P and MraY/C_{55}-P mixtures at pH 8.0 and pH 5.0**.** Peaks corresponding to the dimeric form of UptA is observed mostly in complex with C_{55}-P. **C.** Relative intensity of UptA:C_{55}-P at pH 8.0 and pH 5.0 as a function of concentrations of C_{55}-P in a buffer containing 200 mM ammonium acetate,0.05% LDAO. Data point is an average of 3 independent replicates, and the error bars are standard deviations. **D.** Relative intensity of C_{55}-P (10 µM) bound to UptA (2.5 µM) and MraY (2.5 µM) at different pH. Unlike UptA, there is no pH-dependency in C_{55}-P binding by MraY. Bar represents the mean of 3 replicate measurements shown as data points, and the error bars are standard deviations.

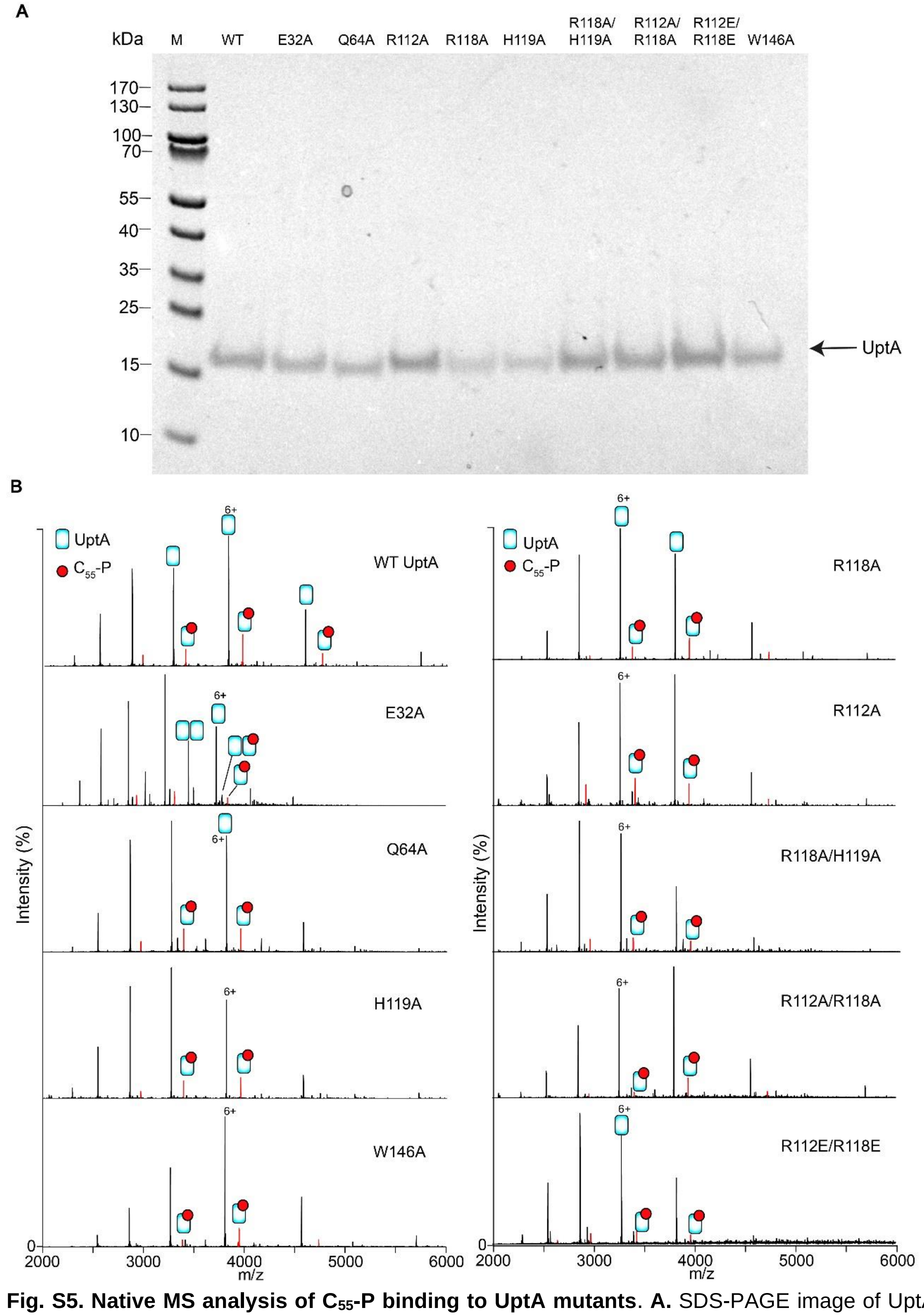

Fig. S5. Native MS analysis of C_{55}-P binding to UptA mutants. **A.** SDS-PAGE image of UptA wild-type (WT) and mutants. M, PageRuler™ prestained protein ladder. UptA is ~22 kDa but migrate on SDS-PAGE as ~17 kDa protein. **B.** Representative spectra for UptA wild type and

mutants (5 µM) incubated with C_{55}-P (10 µM). Proteins were liberated from a buffer containing 0.05% LDAO and 200 mM ammonium acetate (pH 8.02) using a collisional activation of 100 V.

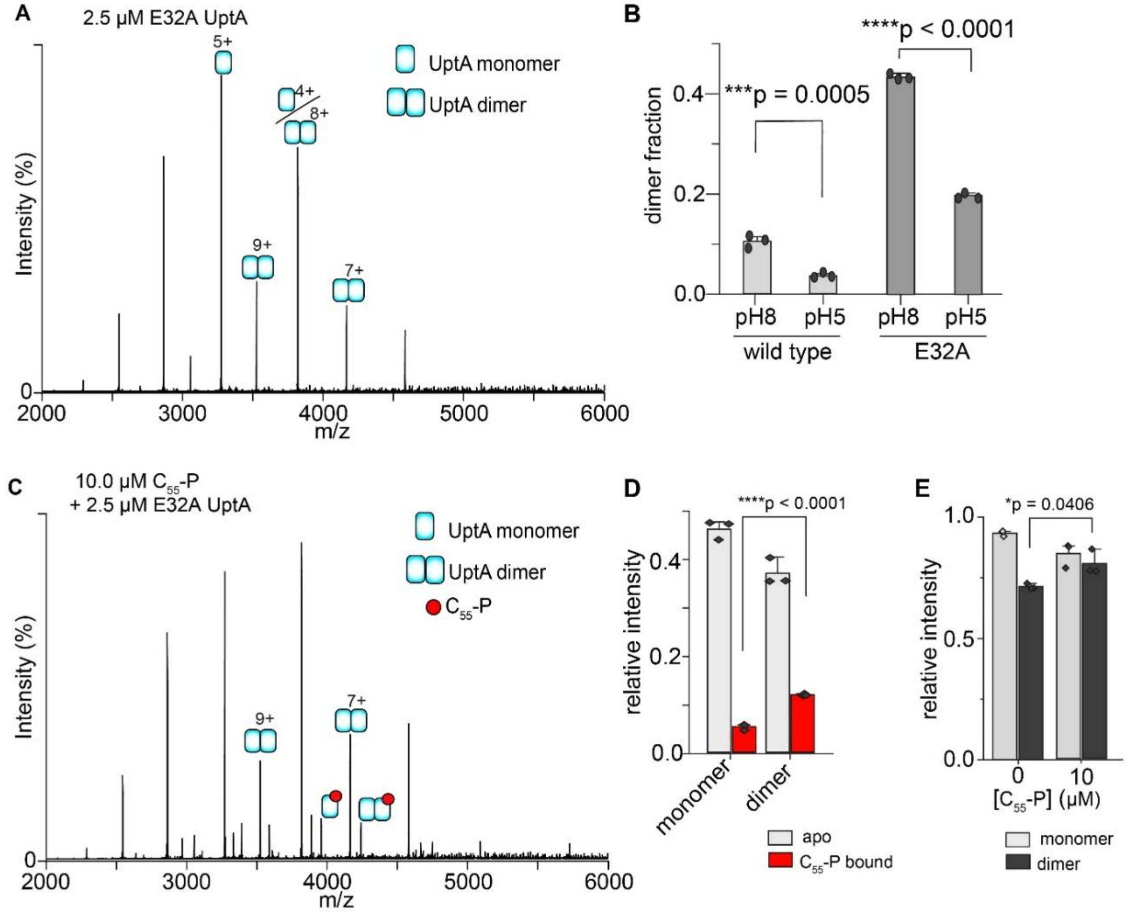

Fig. S6. Dimer fraction of UptA binds C_{55}-P more than the monomeric form. **A.** Spectrum of UptA E32A mutant display peaks corresponding to monomers and dimers. **B.** Relative intensity of dimer populations in spectra for the wild-type UptA and the E32A mutant**.** In both cases, the dimer fraction was higher at pH 8.0 than at pH 5.0. **C.** Spectrum of UptA E32A mutant equilibrated with 10 µM C_{55}-P. **D.** Corresponding intensities of apo and C_{55}-P bound UptA. Dimeric form of UptA bind to the ligand C_{55}-P more significantly than the monomeric form. **E.** The relative proportion of UptA monomers and dimers in a mixture of 2.5 µM E32A UptA and 10 µM C_{55}-P at pH 8.0. The ligand C_{55}-P caused a modest increase in the dimer fraction.

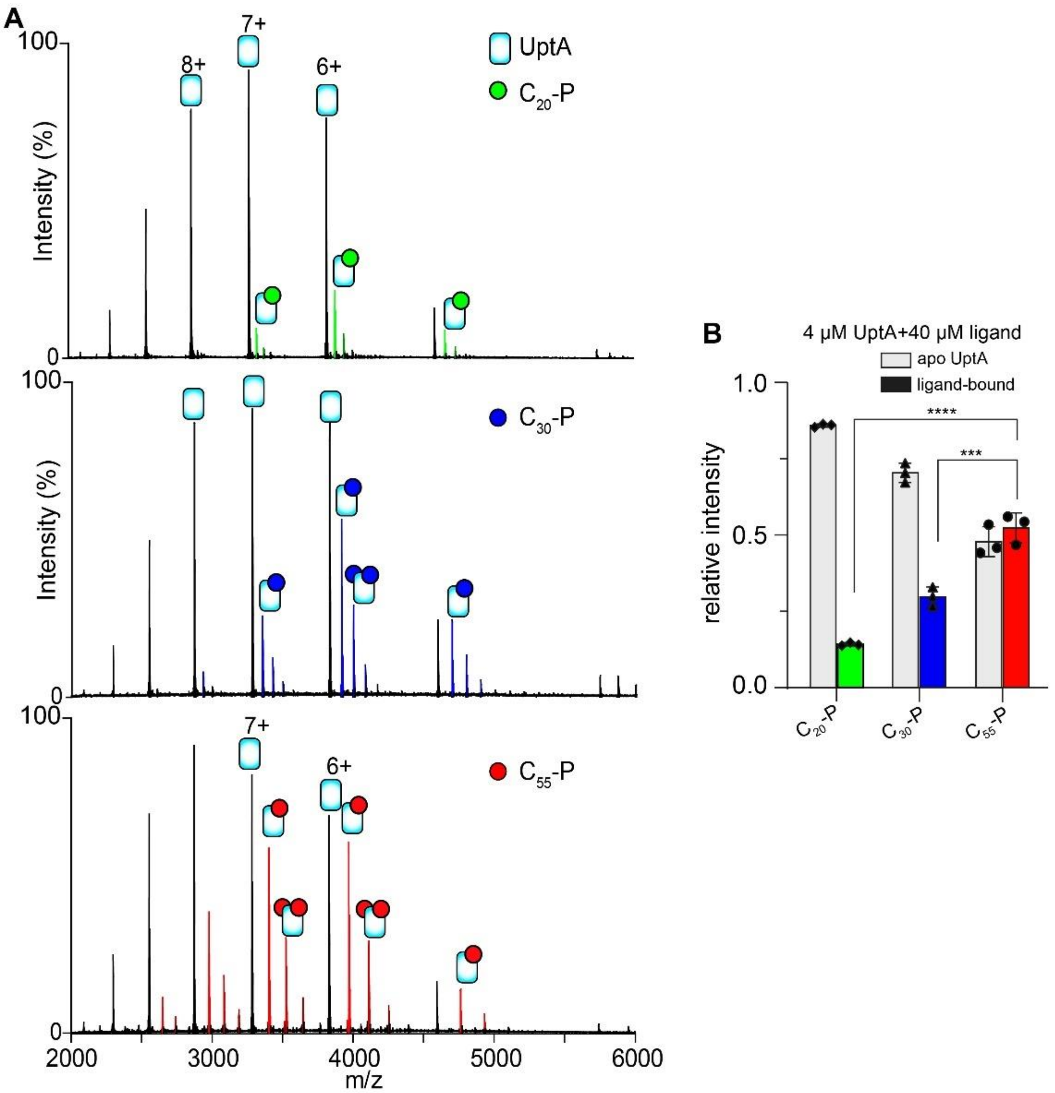

Fig. S7. UptA binds carrier lipids of longer chain lengths more intensely than those of shorter chain, and phospholipids. A. Spectra for 4 µM UptA equilibrated with 40 µM undecaprenyl phosphate, C_{55}-P hexaprenyl phosphate, C_{30}-P and geranylgeranyl phosphate, C_{20}-P. Samples were prepared in a buffer containing 200 MM ammonium acetate (pH8.0) and 0.05% LDAO. **B.** Relative intensity of ligand-free and ligand-bound UptA. UptA binds to C_{55}-P more intensely than the shorter chain analogues.

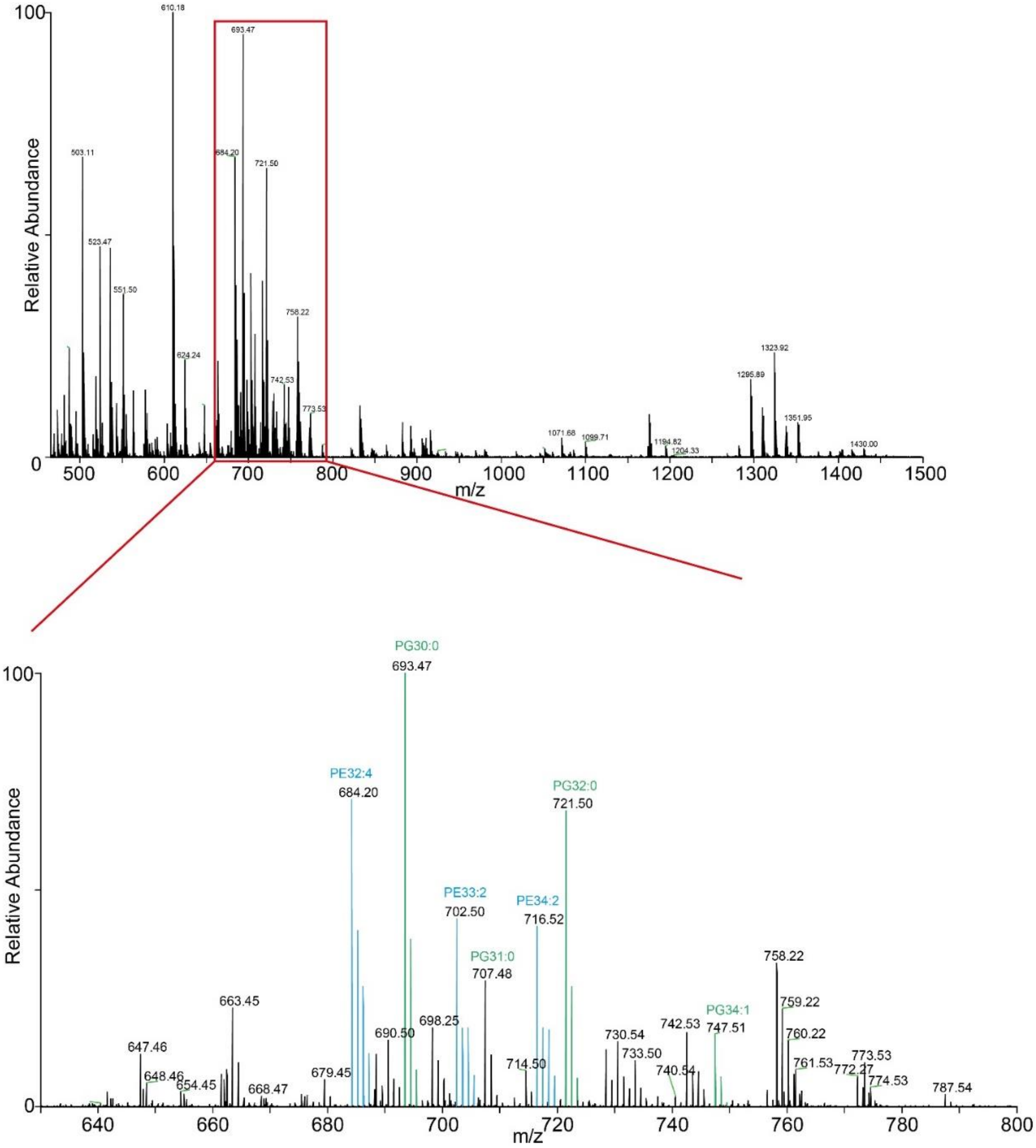

Fig. S8. Mass spectrum of total lipid extract from B. subtilis membrane. Lipid extracts from B. subtilis membrane was dissolved in 0.2 M ammonium acetate, 0.05% LDAO (pH8.0) and analysed in the positive ESI mode using source activation of 25 V. Main phospholipids (PE, blue; PG, green) are highlighted in the expanded view.

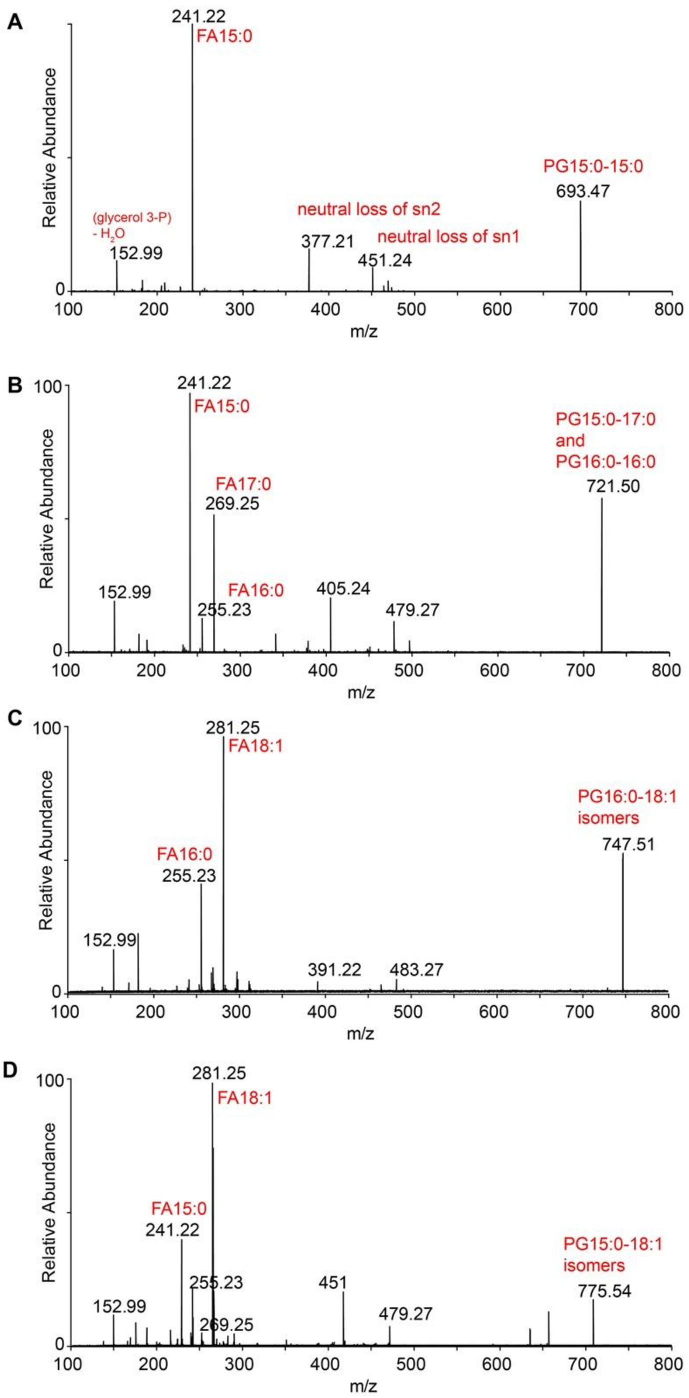

Fig. S9. MS/MS spectra (negative ESI mode) for lipid species interacting with UptA from the total lipid extracts of *B. subtilis*. Peaks corresponding by mass to each of the parent ions were isolated with a source activation of 150 V, and then fragmented with 15-25 V in the HCD cell. All species carry a single negative charged.

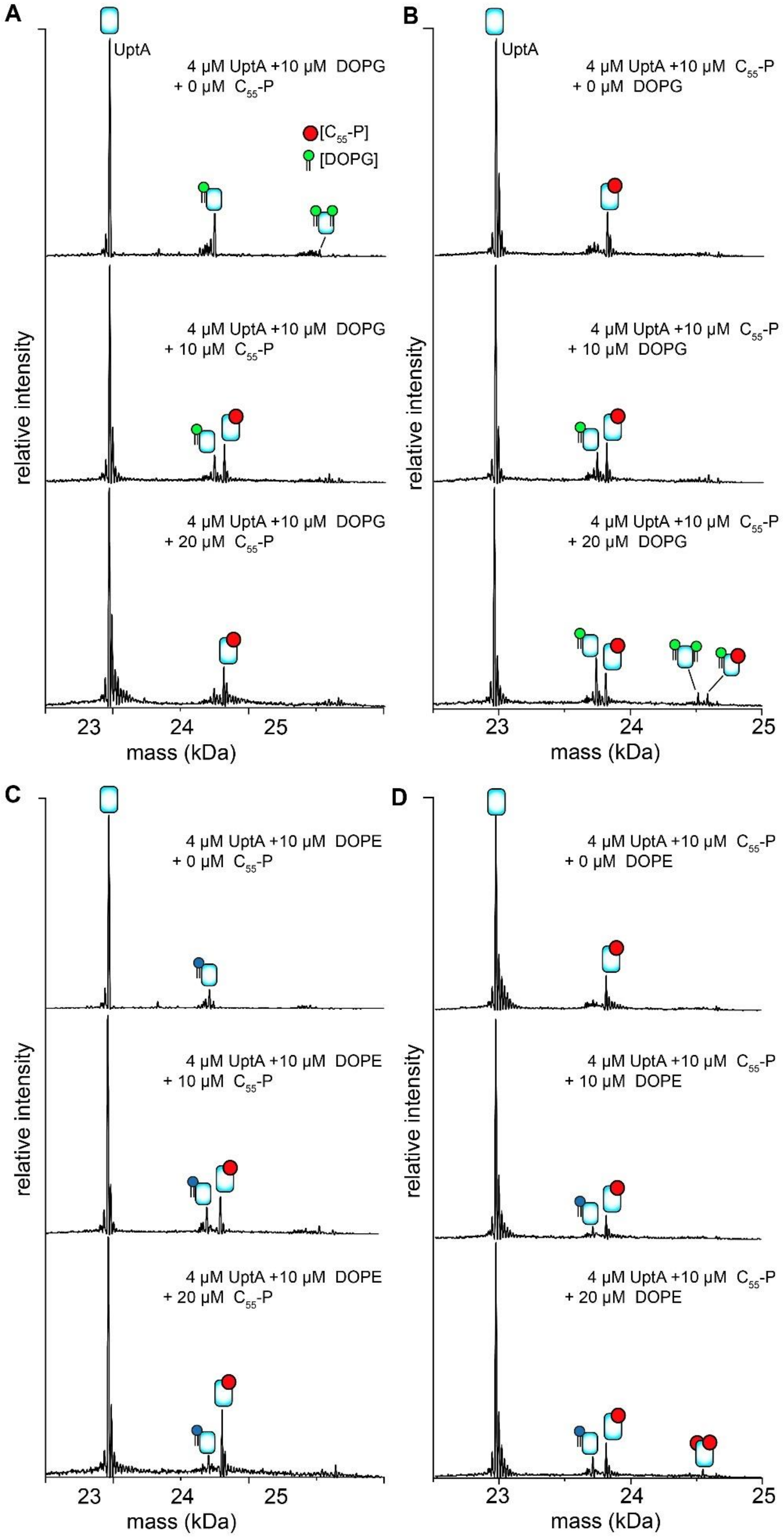

Fig. S10. C55-P outcompetes phospholipids for UptA binding. A,B) Deconvoluted native

mass spectra for 4 µM UptA and10 µM 18:1-18:1 PG (DOPG, panel A) or 18:1-18:1 PE (DOPE, panel B) in the presence of increasing concentrations of C_{55}-P. The intensities of peaks assigned to lipid bound to UptA attenuates upon titration with C_{55}-P. **C,D**) Spectra for 4 µM UptA and 10 µM C_{55}-P, then incubated with increasing concentrations of DOPG (Panel B) or DOPE (panel (D). The peaks assigned to UptA-bound C_{55}-P remained in the presence of lipids, indicating that C_{55}-P binds to UptA more favourable than the phospholipids.

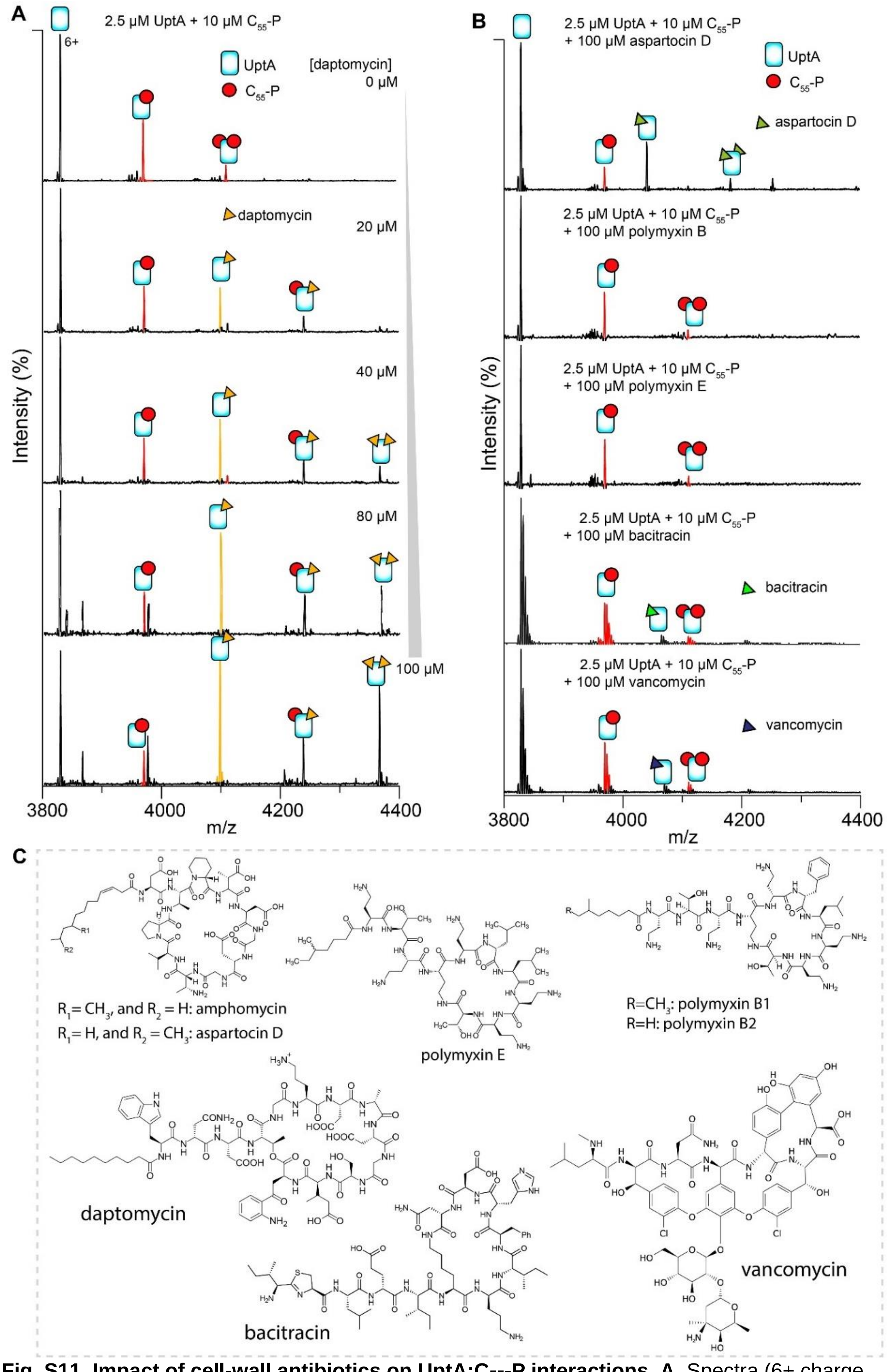

Fig. S11. Impact of cell-wall antibiotics on UptA:C₅₅-P interactions. A. Spectra (6+ charge

state) of 2.5 µM UptA and 10 µM C_{55}-P in the presence of different daptomycin concentrations. Unannotated peaks correspond to multiple daptomycin binding events in the 5+ charge state. **B**. Spectra (6+ charge state) of UptA and C_{55}-P in the presence of different antibiotics and acquired using the same instrument settings and collisional activation of 75 V. Relative intensity of UptA-bound C_{55}-P in the spectra shown in Figure 5. **C**. Chemical structures of lipopeptide antibiotics used in this study.

SI References

1. B. C. Chung *et al.*, Crystal structure of MraY, an essential membrane enzyme for bacterial cell wall synthesis. *Science* **341**, 1012-1016 (2013).
2. A. O. Oluwole *et al.*, Peptidoglycan biosynthesis is driven by lipid transfer along enzyme-substrate affinity gradients. *Nat. Commun.* **13**, 2278 (2022).
3. M. T. Marty *et al.*, Bayesian deconvolution of mass and ion mobility spectra: from binary interactions to polydisperse ensembles. *Anal. Chem.* **87**, 4370-4376 (2015).
4. L. A. Baker *et al.*, Magic-angle-spinning solid-state NMR of membrane proteins. *Methods Enzymol*. **557**, 307-328 (2015).
